# Astrocytic scar restricting glioblastoma via glutamate–MAO-B activity in glioblastoma-microglia assembloid

**DOI:** 10.1186/s40824-023-00408-4

**Published:** 2023-07-19

**Authors:** Yen N. Diep, Hee Jung Park, Joon-Ho Kwon, Minh Tran, Hae Young Ko, Hanhee Jo, Jisu Kim, Jee-In Chung, Tai Young Kim, Dongwoo Kim, Jong Hee Chang, You Jung Kang, C. Justin Lee, Mijin Yun, Hansang Cho

**Affiliations:** 1 Institute of Quantum Biophysics, Sungkyunkwan University, Suwon, 16419 Republic of Korea; 2 Department of Biophysics, Sungkyunkwan University, Suwon, 16419 Republic of Korea; 3 Department of Intelligent Precision Healthcare Convergence, Sungkyunkwan University, Suwon, 16419 Republic of Korea; 4 Department of Nuclear Medicine, Yonsei University College of Medicine, Seoul, 03722 Republic of Korea; 5 Center for Cognition and Sociality, Institute for Basic Science, Daejeon, 34126 Republic of Korea; 6 Department of Biomedical Engineering, Ulsan National Institute of Science & Technology, Ulsan, 44919 Republic of Korea; 7 Department of Neurosurgery, Severance Hospital, Seoul, 120-752 Republic of Korea; 8 Korea University-Korea Institute of Science and Technology, Graduate School of Convergence Technology, Korea University, Seoul, 136-701 Republic of Korea

**Keywords:** Glial scar formation, MAO-B, Glutamate, Glioblastoma, Microglia, Assembloid

## Abstract

**Background:**

Glial scar formation is a reactive glial response confining injured regions in a central nervous system. However, it remains challenging to identify key factors formulating glial scar in response to glioblastoma (GBM) due to complex glia-GBM crosstalk.

**Methods:**

Here, we constructed an astrocytic scar enclosing GBM in a human assembloid and a mouse xenograft model. GBM spheroids were preformed and then co-cultured with microglia and astrocytes in 3D Matrigel. For the xenograft model, U87-MG cells were subcutaneously injected to the Balb/C nude female mice.

**Results:**

Additional glutamate was released from GBM-microglia assembloid by 3.2-folds compared to GBM alone. The glutamate upregulated astrocytic monoamine oxidase-B (MAO-B) activity and chondroitin sulfate proteoglycans (CSPGs) deposition, forming the astrocytic scar and restricting GBM growth. Attenuating scar formation by the glutamate–MAO-B inhibition increased drug penetration into GBM assembloid, while reducing GBM confinement.

**Conclusions:**

Taken together, our study suggests that astrocytic scar could be a critical modulator in GBM therapeutics.

**Graphical Abstract:**

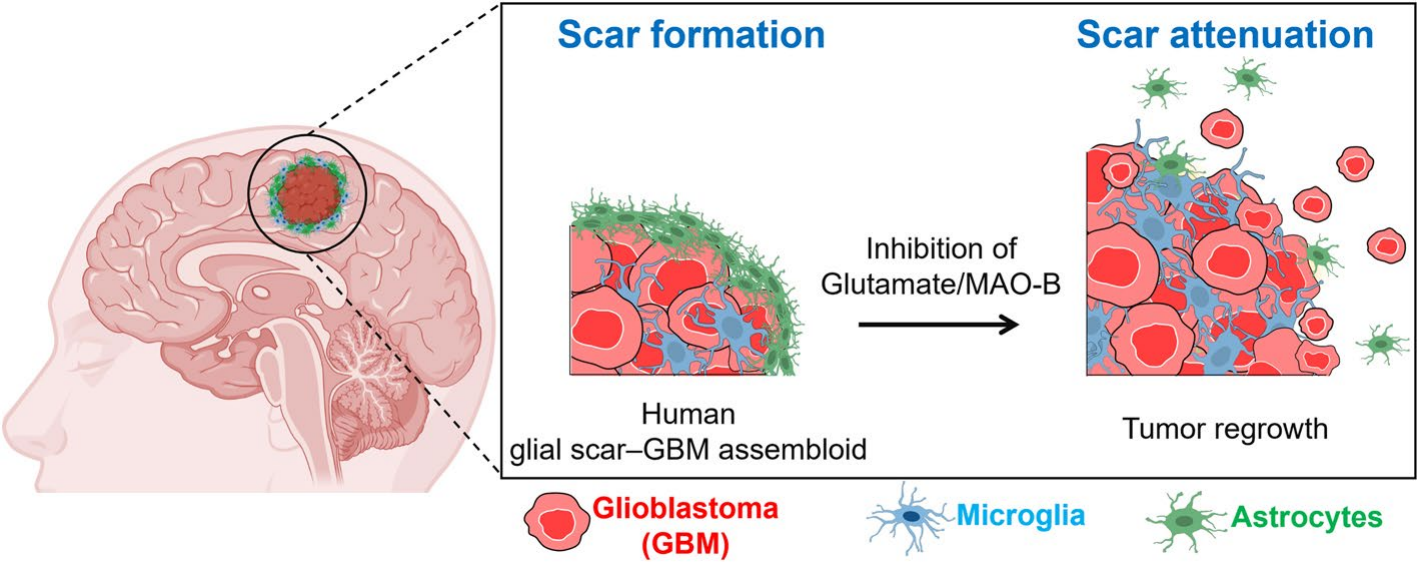

**Supplementary Information:**

The online version contains supplementary material available at 10.1186/s40824-023-00408-4.

## Background

Glial scar formation is a cellular process that occurs after an injury in the central nervous system [1]. Like the scarring process in other tissues and organs, the glial scar formation is a response that initiates the healing process in nervous system. Astrocytes are the major cellular components of glial scar, which become reactive by upregulating the expression of filament proteins such as glial fibrillary acidic protein (GFAP) [2]. Reactive astrocytes also transform into a physical barrier-like structure which isolates the injured regions from surrounding healthy tissue [1]. The chronic astrocytic scar can be accompanied by the increased deposition of extracellular matrix molecules released from reactive astrocytes such as chondroitin sulfate proteoglycans (CSPGs) [3]. In addition to astrocytes, microglia also rapidly accumulate surrounding the lesion borders, which is adjacent to the astrocytic scar [4]. Microglia promote proliferation and activation of scar-forming astrocytes and decrease the inflammatory response upon the injury [5, 6]. There are controversial reports regarding the roles of scar formation. On one hand, formation of a glial scar shows detrimental effects by inhibiting regenerative axons growing pass its border into the injury site [7]. On the other hand, other independent study report beneficial aspect of astrocytic scar by restricting the spread of cytotoxic neuroinflammation and protecting neural tissue adjacent to the scar [8, 9]. The scar formation have been observed not only in the spinal cord injury, but also in other neurological disorders such as glioblastoma multiforme (GBM) [10]. Therefore, it is critical to understand the process of scar formation and its role in the central nervous system.

Monoamine oxidase B (MAO-B) is an enzyme located on the outer membrane of mitochondria [11], which is highly expressed in reactive astrocytes in different types of neuroinflammatory diseases [12, 13]. MAO-B catalyzes biogenic amines and neurotransmitters such as dopamine, resulted in the production of by-product H_2_O_2_ [14, 15]. Recently, it has been demonstrated that H_2_O_2_ produced by MAO-B directly induces astrocytic hypertrophy and scar-forming astrocytes in a stab wound injury mouse model [16]. Notably, it has been reported that inhibition of MAO-B reduces astrogliosis and cellular hypertrophy in neurodegenerative diseases such as Alzheimer’s disease, Parkinson’s disease and subcortical stroke [12, 17, 18]. However, it is not yet fully understood whether MAO-B contributes to astrogliosis and scar formation in glioblastoma or which factors from the tumor microenvironment may be involved in regulating the levels of MAO-B in astrocytes.

Glutamate is an essential excitatory neurotransmitter in the central nervous system. To maintain normal brain function, the extracellular concentration of glutamate is tightly regulated in the range of 1–10 μM by excitatory amino acid transporters (EAATs) of astrocytes [19]. In the glioma microenvironment, glioma cells actively generate a significant amount of glutamate, contributing to the increase of glutamate in and around the tumor mass [20, 21]. A clinical study reported a glutamate concentration of up to 100 μM found in the peritumoral cortex, which is 100-fold higher than that of uninvolved brain tissue [22]. High glutamate level in GBM patients is correlated with seizure and over excitotoxicity in cells adjacent to the tumor sites [23]. However, whether GBM-derived glutamate contributes to astrogliosis and scar formation is unknown.

Here, we established a three-dimensional (3D) human model of astrocytic scar targeting GBM-microglia assembloid and a mouse tumor xenograft model that enabled the mechanism study of astrogliosis and scar formation as well as the role of glial scar in response to GBM. We found that GBM-microglia interplay in the assembloid significantly increased total glutamate level of the GBM microenvironment. We discovered that the excessive glutamate upregulated astrocytic MAO-B expression, leading to astrogliosis, scar formation and confinement of GBM. Inhibiting the glutamate–mediated MAO-B activity in vitro and *in vivo* attenuated the astrocytic scar and increased drug infiltration to the GBM region. Our study suggested that the glutamate–MAO-B activity played a crucial role in astrogliosis and scar formation in response to GBM and has the potential role to confine GBM growth.

## Methods

### Cell culture

Human glioblastoma cell line U87 MG was purchased from American Type Culture Collection (ATCC, Bethesda, MD, USA). The immortalized human microglia and astrocytes SV40 cell line were obtained from Applied Biological Materials, Inc. (ABM, Inc.). GBM was cultured in DMEM/High glucose (SH30243.01, Hyclone) supplemented with 10% Fetal Bovine Serum (FBS) (16000044, Gibco). Microglia was cultured in Prigrow III (ABM-TM003, ABM good) containing 10% FBS. Astrocytes were maintained in Prigrow IV supplemented with 10% FBS, 10 ng mL^−1^ EGF, 2 mM L-glutamine, and 2% penicillin/streptomycin (15070063, Thermo Fisher Scientific). All cells were cultured in T25 flask (70025, SPL Life Sciences) at 37^0^C, 5% CO_2_ incubator. Media were changed every 2–3 days until cells were confluent.

### Primary culture

Primary astrocyte cells were isolated from ICR mice (OrientBio) at postnatal day 1. The cells were seeded on 100 μg mL^−1^ poly-D-lysine (P6407, Sigma-Aldrich) coated plate in DMEM/High glucose media (SH400007.01, Hyclone) supplemented with 10% (wt/vol) FBS (16000–044, Gibco), 10% (wt/vol) horse serum (16050–122, Gibco), 2 mM glutamine (35050, Gibco), 100 U mL^−1^ penicillin/streptomycin (15140–122, Gibco). After 7 days, attached non-astrocytic glial cells were removed by shaking vigorously and then astrocyte cells were cultured in DMEM/High glucose media (SH400007.01, Hyclone) supplemented with 10% (wt/vol) horse serum (16050–122, Gibco), 2 mM glutamine (35050, Gibco), 100 U mL^−1^ penicillin/streptomycin (15140–122, Gibco).

### Primary human GBM culture

The use of human tissue samples was approved by the Institutional Review Board at Yonsei University Health System Severance Hospital (Seoul, Korea, Yonsei IRB number 4–2019-0455). This study was conducted according to the current guidelines for ethical research. All patients provided oral and written consent after receiving detailed information on the study and agreed to data collection. Primary human GBM cells were isolated using the brain tumor dissociation kit (130–095-942, Miltenyl Biotec). Briefly, the tumor specimen was dissociated by Kit’s enzyme and incubated on the gentleMACS dissociator at 37 °C. Then, tissues were filtered through a 7-μm smart-strainer and cells were collected by centrifugation. The tumor cell spheres were cultured in non-adherent plates using EMEM media supplemented with 2 mM L-glutamine, 2% penicillin/streptomycin, 20 μL mL^−1^ B27, EGF 20 ng mL^−1^, and 20 ng mL^−1^ FGF.

### Fabrication of the *in vitro* GBM-microglia assembloid with astrocytic scar model

GBM cells were harvested with Trypsin–EDTA (25200056, Thermo Fisher Scientific) and 100 μL of GBM cell suspension (cell seeding density of 10^4^ cells mL^−1^) were seeded into each well of the round-bottom ultra-low attachment microplate (4515, Corning). The GBM cells were incubated for 24 h at 37^0^C, 5% CO_2_ for spheroid formation. The U87-MG spheroids or patient-derived GBM spheres were transferred to a flat-bottom 96 well plate, and co-cultured with 50 μL glia cell suspension in Matrigel. The glia/Matrigel suspension (total cell seeding density of 6 × 10^5^ cells mL^−1^) composed of microglia (cell seeding density of 75 × 10^4^ cells mL^−1^), astrocytes (cell seeding density of 75 × 10^4^ cells mL^−1^), and Matrigel with 2:2:1 (v/v/v) ratio, respectively. After 1-h polymerization of Matrigel, 100 μL media were added to each microwell. To fabricate the GBM MG or GBM AC models, GBM spheroids were co-cultured with 50 μL of microglia (cell seeding density of 75 × 10^4^ cells mL^−1^) or astrocytes (cell seeding density of 75 × 10^4^ cells mL^−1^) in Matrigel, respectively, with cells: Matrigel in 4:1 (v/v) ratio. GBM assembloids were cultured in serum-free media composing EMEM, 2 mM L-glutamine, 2% penicillin/streptomycin, freshly supplemented with 20 μL mL^−1^ B27, EGF 20 ng mL^−1^, and 20 ng mL^−1^ FGF prior using [24]. The 3D in vitro models were cultured at 37^0^C, 5% CO_2_.

### Mouse glioma model

All experiment policies and procedures were approved by the Institutional Animal Care and Use Committee of Yonsei University College of Medicine. The 5 weeks age of Balb/C nude female mice were purchased from OrientBio and maintained at 20–22 °C in a 12 light/dark cycle. For the orthotopic glioma model, mice were anesthetized with 2% isoflurane during the procedure. U87 MG cells (2 × 10^5^ cells μL^−1^) were prepared and 2 μL of suspended cells were injected (injection site: AP + 0.5; ML -2; DV -3 to bregma) using a stereotaxic device. This study did not have humane endpoints and there were no unexpected events happened to the mice.

### Quantification of the GBM area

U87 MG cells or patient-derived GBM spheres were labeled with red fluorescent dye (PKH26, Sigma-Aldrich). Cells were resuspended in 0.5 mL Diluent C, then mixed with 4 μL red dye solution dispersed in 0.5 mL Diluent C. The cell-dye mixture were incubated at room temperature for 5 min. After incubation, 4 mL phosphate-buffered saline (PBS) were added to the mixture and centrifuged to remove unbound dye. Patient-derived GBM spheres or GBM spheroids formed by U87 MG-red labeled cells were imaged using TRITC channel (Eclipse Ti2-E, Nikon). The area of spheroids was automatically determined by the Auto Detection ROI tool in NIS-Elements Advanced Research software.

### Quantification of the scar-forming astrocytes/microglia

Astrocytes were labeled with green fluorescent protein (GFP). Microglia were stained with Cy5 fluorescent dye (MIDCLARET, Sigma-Aldrich) using the protocol similar to that of U87 MG labeling described above, however, the Diluent C was replaced by Diluent B. Upon the microgliosis and astrocytic scar formation, confocal images were taken and the number of microglia/astrocytes from the middle plane of confocal images were calculated by ImageJ (NIH, Bethesda).

### Human cytokine assay

Upon GBM-microglia assembloid model construction, 1 mL of conditional media were collected for cytokine detection. The relative expression of cytokines were assessed by the human cytokine array kit following the manufacturer’s protocol (ARY005B, R&D systems). Briefly, the nitrocellulose membranes printed with anti-cytokines antibodies were blocked with Array Buffer 4. After blocking, the membranes were incubated overnight at 4^0^C with the sample/antibody mixture containing conditional media, detection antibody cocktail, and array buffer 4. After washing thoroughly, the membranes were incubated with streptavidin-HRP diluted in Array Buffer 5. The membranes were washed again, followed by the incubation with Chemi Reagent Mix. After incubation, the chemiluminescence were detected and the pixel density of signals were analyzed by ImageJ (NIH, Bethesda).

### Glutamate determination

The glutamate level from conditional media were measured by the glutamine/glutamate determination kit (GLN1, Sigma-Aldrich) following the protocol of manufacturer. Briefly, we prepared a mixture composing Tris–EDTA-hydrazine monohydrate, adenine dinucleotide (NAD), and 5’-diphosphate (ADP) in 100:10:1 (v/v/v) ratio. This mixture was mixed with Glutamate Standards (for standard wells) or samples (for sample wells). The absorbance was measured by the spectrophotometer at 340 nm wavelength to obtain the background reading. After measurement, the glutamic dehydrogenase (L-GLDH) was added to the standard and sample wells and incubated at room temperature for 40 min. The absorbance was read again with the spectrophotometer and was subtracted to the background absorbance to obtain net absorbance.

### Assessment of the astrocytic CSPGs

Astrocytes were seeded to 96 wells coated with collagen I (354249, Corning) at a density of 1.5 × 10^4^ cells/well. After 24 h, the astrocytes were treated with 400 μM glutamate in media supplemented with 2% FBS. 50 μL media were used to perform the ELISA following the previously published protocol [19].

### H_2_O_2_ measurement

To detect the presence of H_2_O_2_, one type of reactive oxygen species (ROS), we incubated astrocytes with 10 μM ROS indicator (D399, Thermo Fisher Scientific) for 30 min at 37^0^C, 5% CO_2_. After the incubation, the astrocytes were washed three times with prewarmed astrocytes media. The fluorescence intensity were detected using inverted microscope (Eclipse Ti2-E, Nikon).

### Immunocytochemistry

PBS (PR4007-100–74, Biosesang) supplemented with 0.1% w/v bovine serum albumin (BSA100, Bovogen) and 0.1% v/v Tween 20 (9005–64-5, Sigma-Aldrich) was used as washing and dilution buffer. After rinsing twice with PBS, cells were fixed with fresh 4% paraformaldehyde solution (PFA, PC2031-100–00, Biosesang) at room temperature for 20 min, and were washed three times with the washing buffer. Then, cells were permeabilized using PBS supplemented with 0.1% v/v Tween 20 and 0.1% v/v Triton X-100 (9036–19-5, Sigma-Aldrich) for 30 min at room temperature and were washed three times with the washing buffer. Next, the cells were incubated in the blocking solution (PBS supplemented with 0.1% v/v Tween 20 and 3% w/v BSA) for 1 h at room temperature. After overnight incubation at 4^0^C with the primary antibody and rinsing three times with the washing buffer, cells were stained with secondary antibody for 1.5 h at room temperature. Cell nuclei were stained with Hoechst (33342, Thermo Fisher Scientific).

### Immunohistochemistry for mouse tissues

Perfused mouse brains were fixed in 4% PFA and immersed in 30% sucrose solution until sinks. Then, the brains were embedded with pre-chilled OCT solution (3801480, Leica). Frozen tissues were sectioned using a cryostat. Coronal sections were incubated with blocking solution (0.3% Triton X-100 and 5% normal serum in PBS) and immunostained with a mixture of primary antibodies diluted in blocking solution at 4 °C on a shaker overnight. Then, the sections were rinsed three times with PBS and stained with secondary antibody for 1 h at room temperature. Cell nuclei were stained with Hoechst (33342, Thermo Fisher Scientific) or DAPI (H-1200, VECTASHIELD).

### Immunohistochemistry for human tissues

Human tissue was fixed with 4% PFA, made into paraffin blocks, and cut into 4 μm sections. After antigen retrieval, the slides were blocked with the blocking solution and incubated with antibodies as described above.

The following primary and secondary antibodies (dilutions, Cat. No., Company) were used for immunostaining: anti-CD86 (1:100, ab201340, Abcam), anti-CD206 (1:200, NB6001415, Novus Biological), anti-Iba (1:100, MABN92, Millipore), anti-GFAP (1:500, AB5541, Sigma-Aldrich and 1:1000, Z0334, Dako), anti-Ki67 (1:50, M7240, Dako), anti-chondroitin sulfate (1:500, C8035, Sigma-Aldrich), anti-GLUT1 (1:100, ab40084, Abcam), Alexa Fluor 647 goat anti-chicken (1:500, ab150171, Abcam), Alexa Fluor 594 goat anti-rabbit (1:500, ab150080, Abcam), Alexa Fluor 647 goat anti-mouse (1:500, ab150115, Abcam).

### Western blot analysis

The cells were lysed with RIPA lysis buffer (89900, Thermo Fisher Scientific) containing protease inhibitor (11836153001, Roche) and total protein concentration were determined by BCA protein assay kit (23225, Thermo Fisher Scientific). Protein lysates were separated by 10% of SDS-PAGE and transferred to a PDVF membrane. The following primary and secondary antibodies (dilutions, Cat. No., Company) were used: anti-MAO-B (1:1000, NBP1-87493, Novus Biological), anti-EAAT1 (1:1000, AB181036, Abcam), anti-Actin (1:1000, SC-47778, Santa Cruz), goat anti-rabbit (1:2000, GTX213110, GeneTex), goat anti-mouse (1:2000, GTX213111, GeneTex).

### Assessment of the small molecular infiltration *in vitro*

Upon the glial scar formation (day 4), we added 0.5 μg mL^−1^ fluorescein sodium salt NaFI (518–47-8, Sigma-Aldrich) diluted in media to the cell culture models and incubated the cells at 37^0^C for 24 h. After the incubation, cells were washed 3 times with media and were immediately imaged to obtain the NaFI fluorescence intensity. To assess the infiltration of NaFI molecular into the GBM spheroid area, we determined the ROI for quantifying NaFI intensity same as that of GBM’s ROI, which was described above.

### Drug treatment

Glutamate uptake and MAO-B expression in astrocytes were inhibited using glutamate transporter inhibitor TBOA (1223, Tocris) (100 μM for *in* *vitro* model, 30 mg/kg/day for *in vivo* model) and our newly developed MAO-B inhibitor KDS2010 (1 μM for *in* *vitro* model, 10 mg/kg/day for *in vivo* model) [25], respectively. In addition, 500 μM TMZ (T2577, Sigma-Aldrich) was used as chemotherapy drug. PBS 1X (PR4007-100–74, Biosesang) was used as control to TMZ.

### *Invitro* imaging and analysis

Images were captured by using the inverted microscope (Eclipse Ti2-E, Nikon) or confocal laser scanning microscope (LSM 710, Zeiss). Images were analyzed by NIS-Elements Advanced Research imaging software and ImageJ (NIH, Bethesda).

### Magnetic resonance imaging (MRI) and analysis

MRI examinations were performed on a 9.4 T BrukerBioSpin MR scanner (GmbH, Germany). Acquistion parameters were as follows: TR = 1800 ms, TE = 24 ms, flip angle = 90°, FOV = 20 × 20 mm, 192 × 192 matrix with 0.104 × 0.104 mm spatial resolution, 20 axial slices and a slice thickness of 0.5 mm for 2D T2-weighted Turbo-RARE sequences. Regions of interest (ROIs) of tumor were outlined on every slice of the 3D MR images, and the tumor volume was calculated based on these ROIs using PMOD v3.5 software (PMOD technologies Ltd., Zurich, Switzerland).

### Assessment of drug penetration *invivo*

One week after the injection of tumor cells, tumor formation was examined by T2-weighted MRI. Tumor-bearing mice were randomly divided into four groups and allowed to drink water (control) or KDS2010 (10 mg/kg daily) for 1 day, 3 days and 7 days. Two weeks after injection of tumor cells, 10 mM dosage of Doxorubicin (DOX, 44583, Sigma-Aldrich) was intravenously administered and the intracardiac perfusion was performed 30 min after injection. Optical fluorescence signals of DOX were obtained using IVIS imaging system (Caliper Life Sciences) with excitation wavelength of 500 nm and emission wavelength of 600 nm.

### Assessment of drug sensitivity *invivo*

Balb/c nude mice (female, 5 weeks old, LaonBio Inc) were anesthetized with 2% isoflurane and oxygen. 3 mg D-luciferin solution (122799, PerkinElmer) was intraperitoneally injected to each mouse. Images were taken by IVIS® Lumina III In Vivo Imaging System (PerkinElmer) analyzed with Living Imaging software v.4.7.4 (PerkinElmer).

### Statistical analysis

Data analysis were performed using Graphpad Prism 8 software (GraphPad Software, La Jolla, CA). Two-tailed unpaired Student’s t-test was used to compare two groups/conditions. Turkey post-hoc one-way ANOVA was used to compared multiple groups/conditions. Difference in survival rate was analyzed using log-rank (Mantel-Cox) test. Data were presented as mean ± standard deviation (SD) except data in Fig. 5j which was presented in mean ± Standard Error of Mean (SEM). *p* ≤ 0.05 was statistically considered significant. The *, **, ***, ****, ns represented *p* < 0.05, *p* < 0.01, *p* < 0.001, *p* < 0.0001, and no significance, respectively. No statistical techniques were performed to predetermine optimal sample size, but experiments were adequately repeated to minimize confidence.intervals and errors in statistical tests. Data collection/analysis were not blindly performed to the experimental conditions. Exclusions were not performed, and randomization was not made. All statistical analyses, including groups being compared and control groups, were detailed in the figure legend and were summarized in Additional file 1: Supplementary Table 1.  All outcome measures were summarized in Supplementary Table 2, Additional file 2.

## Results

### Construction of glial scar-GBM *in vitro *and* invivo* models

**Fig. 1:**
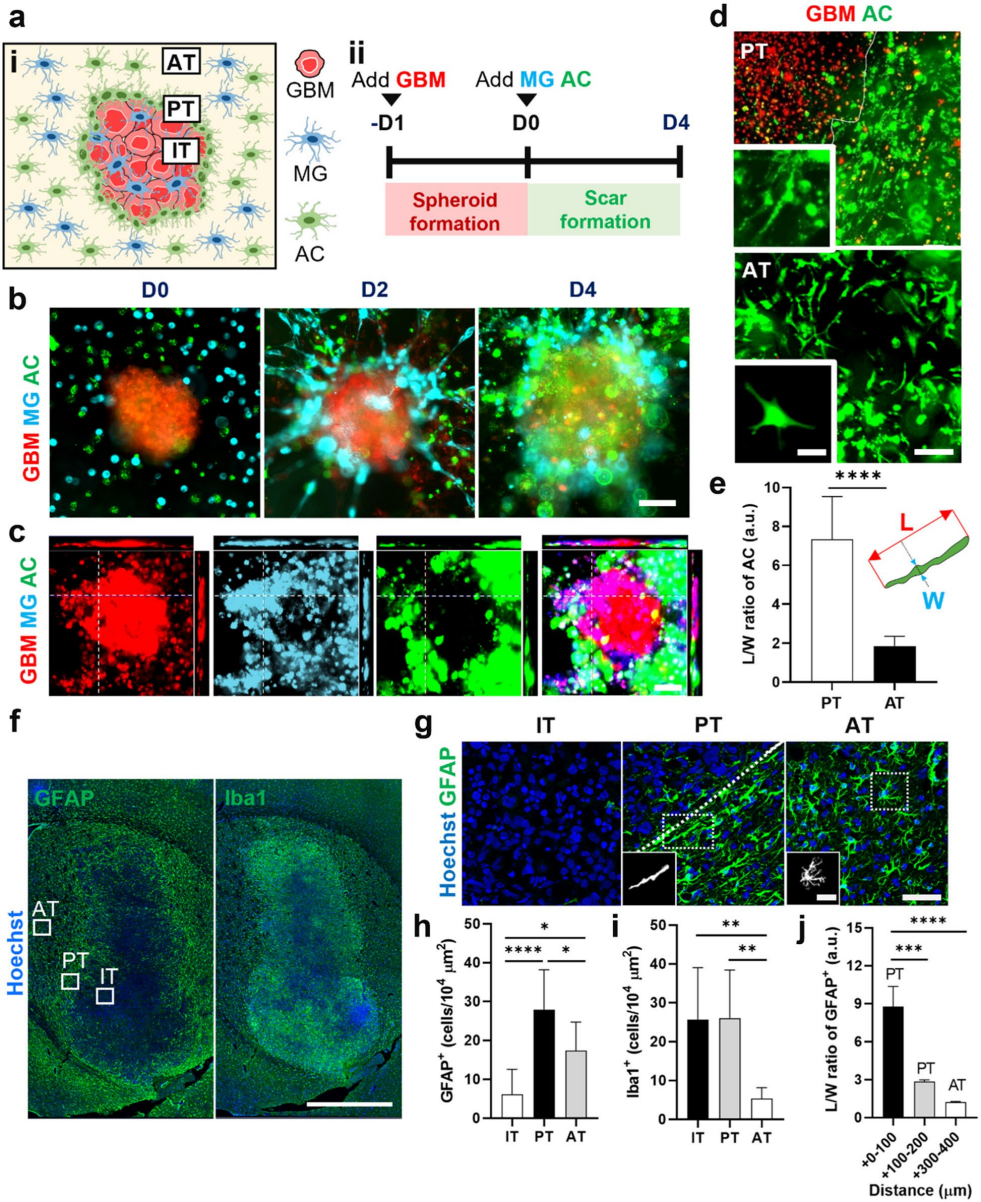
Construction of glial scar-GBM in vitro and *in* *vivo* models. **a** i) Schematic representing the distribution of astrocytes and microglia in the scar formation. ii) Timeline for the in vitro glial scar-GBM assembloid construction. **b** Time-lapse images of the glial scar formation in vitro. **c** Confocal images of the assembloid showing astrocytic and microglial distribution. **d** Morphology of astrocytes at the PT and AT region of the GBM assembloid. **e** Length/width (L/W) ratio of astrocytes at the PT and AT region (*n* = 10). **f** Distribution of astrocytes (GFAP^+^) and microglia (Iba1^+^) in accordance with GBM *in* *vivo*. **g** Morphology of astrocytes at the IT, PT and AT regions of *in vivo* model. **h**-**i** Quantification of GFAP^+^ and Iba1^+^ cells in the IT, PT, and AT regions (*n* = 9). **j** L/W ratio of GFAP^+^ cells in the PT and AT regions (*n* = 10). Quantitative data were presented as means ± SD (*n* = 3, unless otherwise noted). *, *p* < 0.05; **, *p* < 0.01; ***, *p* < 0.001; ****, *p* < 0.0001. *p* values were calculated by two-tailed unpaired Student's t-test. Scale bars represent 100 μm (**b**, **c**, **g**), 25 μm (**d**, **g** insets), 200 μm (**d**), 1000 μm (**f**). IT, intra-tumor; PT, peri-tumor; AT, away from tumor; GBM, glioblastoma; MG, microglia; AC, astrocytes

We developed an in vitro GBM-microglia assembloid with astrocytic scar by co-culturing GBM spheroids with glial cells in Matrigel. Schematic illustration of the glial scar formation responding to GBM and timeline for the model construction were summarized in Fig. 1a and Supplementary Fig. 1. Briefly, GBM spheroids were pre-formed and then co-cultured with astrocytes and microglia in 3D Matrigel. Through this method, we observed that microglia and astrocytes, which homogenously distributed surrounding GBM spheroids on day 0, were gradually accumulated around GBM spheroids over 4 days of co-culture (Fig. 1b). We replicated this observation using serum-free media and confirmed compatible results with media containing 10% FBS (Supplementary Fig. 2a). Next, we performed the confocal imaging to further investigate the distribution of astrocytes and microglia in accordance with GBM spheroids. The middle plane of confocal images showed that microglia were presented not only at the interface of GBM spheroids but also inside the spheroids, while the majority of astrocytes only accumulated around GBM spheroids (Fig. 1c). Previous study has reported the morphological changes of astrocytes to elongated shape in mature astrocytic scar border [26]. To explore the astrocytic morphology, we cultured GBM spheroids and astrocytes in Matrigel for 4 days and found that astrocytes became reactive, shown by an increase in GFAP expression (Supplementary Fig. 3). As diameter of the glial scar-GBM was approximately 500 μm, we determined the 500-μm diameter circle with center point same as that of GBM spheroids was the area for quantification of scar-forming glial cells, called as scar-GBM circle (Supplementary Fig. 4). We defined the region outside the GBM but within the scar-GBM circle as peri-tumor (PT) region and the region outside the scar-GBM circle as away from tumor (AT) region. We observed that astrocytes at the PT region formed elongated shape, while astrocytes at the AT region maintained their star shape (Fig. 1d). The morphology of astrocytes was quantified by the ratio between length (L) and width (W). Length is determined by the longest dimension of a cell, and width is defined by the longest dimension that is perpendicular to the length. The L/W ratio of astrocytes at PT and AT region were 7.3 and 1.9, respectively (Fig. 1e).

To develop an *in* *vivo* glial scar-GBM model, we injected U87 MG cells to the nude mice. After 28 days, we performed immunostaining for brain tissues with GFAP and Iba1 to assess morphology/distribution of astrocytes and microglia, respectively (Fig. 1f). Based on morphology of astrocytes, we determined the region from tumor margin to margin + 200 μm as PT region (Supplementary Fig. 5). Region within GBM tumor and region with margin +  > 200 μm was determined as intratumor (IT) and away from tumor (AT) region, respectively (Supplementary Fig. 5). We counted number of cells per 100 × 100 μm area at multiple regions (IT, PT, AT) to explore the cell density and distribution. We found a significant increase of GFAP^+^ astrocytes at PT region (27.9 ± 10.3) compared to AT region (17.3 ± 7.3), while we counted only an average of 6.1 GFAP^+^ cells at the IT region (Fig. 1g, h). However, we observed a similar distribution of Iba1^+^ microglia at IT region (27.4 ± 13.1) and PT region (27.6 ± 12.2), and the number of microglia significantly decreased to an average of 5.5 cells at AT region (Fig. 1i). In addition, we observed that astrocytes at the PT region transformed their morphology to elongated shape while astrocytes at the AT region maintained their star shape (Fig. 1g), which was compatible to the observation on *in vitro* model. The L/W ratio of astrocytes in the region from tumor margin to tumor margin + 100 μm was 8.8, decreasing to 1.3 at the tumor margin + 300–400 μm region (Fig. 1j). It should be noted that IT region also expressed GFAP as we used U87 MG cell line (ATCC) for the xenograft mouse model, however, their intensity was significantly lower compared to that of PT (Supplementary Fig. 6). These results indicated the accumulation of both astrocytes and microglia at the interface of GBM, where they transformed their morphology (astrocytes) or infiltrated into the GBM tumor mass (microglia). In sum, we constructed functional and physiologically relevant in vitro and *in* *vivo* models mimicking the glial scar formation responding to GBM.

### GBM and microglia interplay leading to glutamate upregulation

**Fig. 2:**
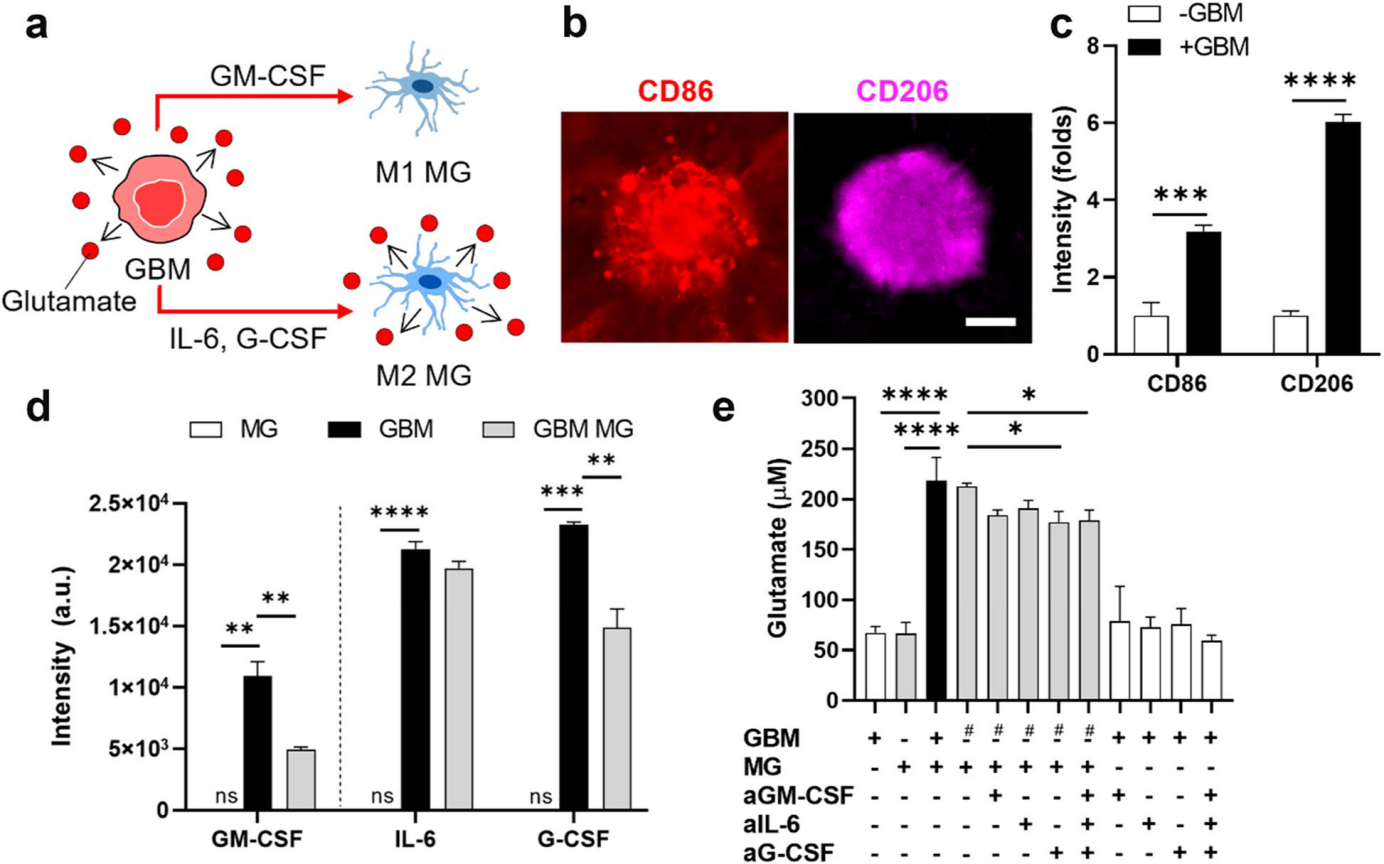
GBM and microglia interplay leading to glutamate upregulation. **a** Schematic representation of GBM and microglia interplay. **b**, **c** Microglial phenotypes in the GBM-microglia assembloid assessed by immunostaining with CD86 (M1) and CD206 (M2) markers. Scale bar represents 200 μm. **d** Cytokine profiles of GBM and microglia. GBM-derived cytokines, GM-CSF inducing microglial polarization to M1 phenotype and IL-6 and G-CSF to M2 phenotype were detected (*n* = 2). **e** Increase of glutamate level mediated by GBM-microglia interplay. Adding neutralizing antibodies targeting GM-CSF (aGM-CSF), IL-6 (aIL-6), and G-CSF (aG-CSF) in the GBM conditional media decreased glutamate level released from microglia. # indicates microglia single culture treated with GBM conditional media. Quantitative data were presented as means ± SD (*n* = 3, unless otherwise noted). *, *p* < 0.05; **, *p* < 0.01; ***, *p* < 0.001; ****, *p* < 0.0001. *p* values were calculated by two-tailed unpaired Student's t-test for comparisons between two groups, and by one-way ANOVA for multiple comparisons. GBM, glioblastoma; MG, microglia

Recent study have reported that the microglial response upon spinal cord injury promoted proliferation and reactivity of astrocytes in the glial scar [5, 6]. Thus, we explored the role of microglia for the formation of astrocytic scar in GBM microenvironment. To this end, we first assessed the microglial reactivity in GBM-microglia assembloid by immunostaining with M1 (CD86) and M2 (CD206) marker. We found both M1 and M2 microglial population in the assembloid (Fig. 2b, c). We also detected cytokines (GM-CSF, IL-6, and G-CSF) dominantly released from GBM, while no significant amount of these cytokines were detected from microglia (Fig. 2d). These results suggested that GBM released cytokines to polarize microglia to M1 (by GM-CSF) and M2 phenotype (by IL-6 and G-CSF). In addition, we detected a significant increase of glutamate (3.2-fold) from GBM-microglia assembloid compared to GBM or microglia single culture (Fig. 2e). To investigate whether additional glutamate from GBM-microglia assembloid released by either microglia or GBM cells, we neutralized cytokines in the GBM conditional medium using neutralizing antibodies targeting GM-CSF (aGM-CSF), IL-6 (aIL-6), and G-CSF (aG-CSF) and treated the neutralized conditional medium on single-culture microglia. We found a decreased glutamate level released from microglia treated with neutralized conditional medium compared to that of non-neutralized medium (Fig. 2e), suggesting the role of these cytokines in promoting glutamate release from microglia. However, treatment of these neutralizing antibodies for GBM single culture did not decrease the average glutamate level compared to that of non-treated GBM (Fig. 2e). These results suggested the contribution of these cytokines to the glutamate increase mediated by GBM-microglia interplay in the assembloid. Proposed mechanism for the role of microglia was summarized in Fig. 2a. GBM-microglia crosstalk via GM-CSF, IL-6 and G-CSF cytokines led to the increase of total glutamate level in the tumor microenvironment.

### Glutamate-driven astrocytic MAO-B leading to reactive astrogliosis and astrocytic scar formation

**Fig. 3:**
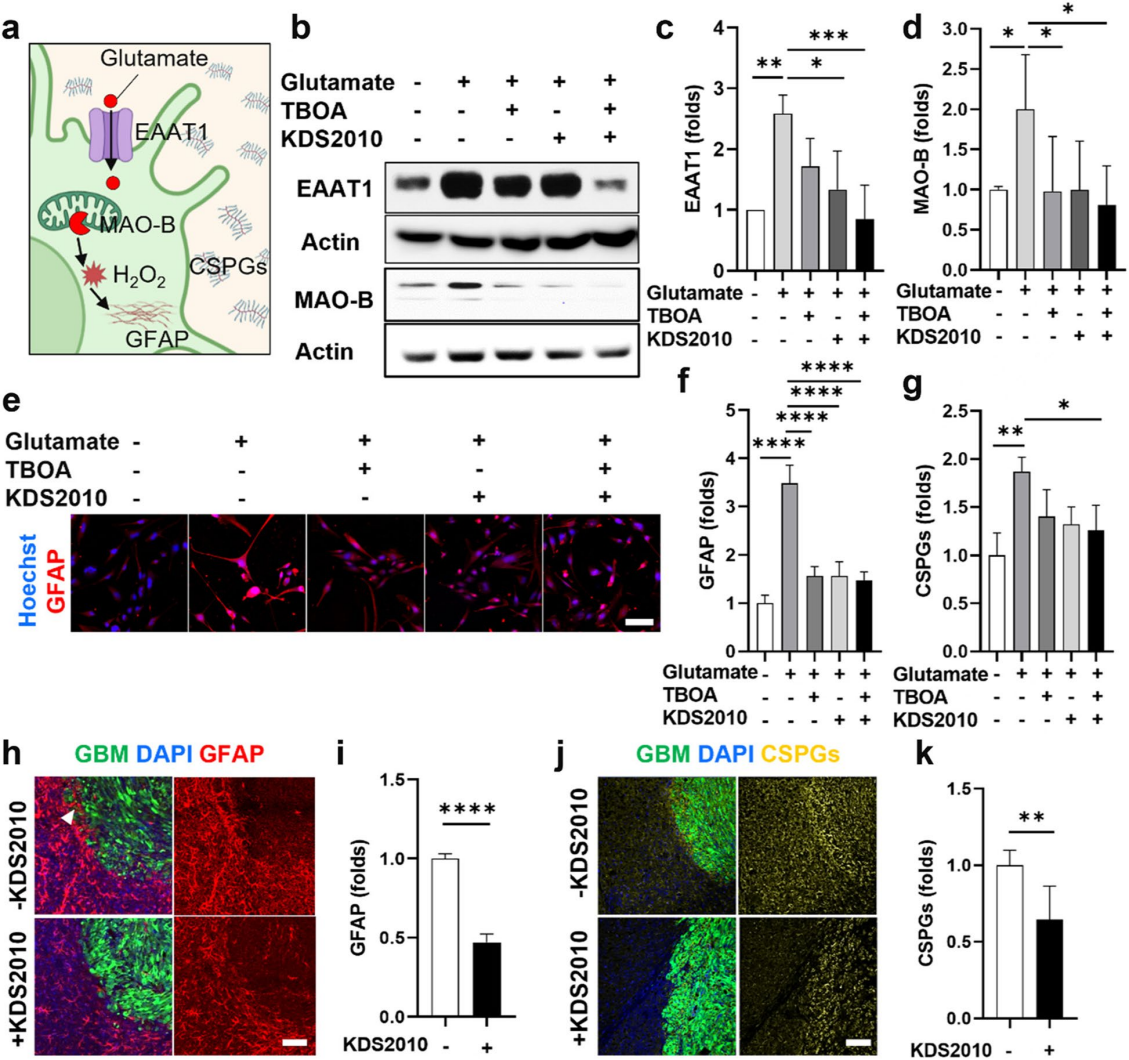
Glutamate-driven astrocytic MAO-B leading to reactive astrogliosis and astrocytic scar formation. **a** Schematic representation of glutamate-driven astrocytic MAO-B inducing reactive astrogliosis and astrocytic scar formation. **b**-**d** Western blot analysis for EAAT1 and MAO-B expression in astrocyte monocultures upon glutamate treatment. Glutamate treatment increased MAO-B expression in astrocytes. Blockage of glutamate transport activity by using glutamate transporter inhibitor (TBOA) decreased MAO-B expression. **e**, **f** Reactivity of astrocyte monocultures upon glutamate treatment assessed by immunostaining with GFAP. Inhibiting glutamate transport (TBOA) or MAO-B (KDS2010) decreased GFAP expression. **g** ELISA for CSPGs deposited from reactive astrocytes. **h**, **i** Immunofluorescence images and quantification of GFAP intensity in the mouse tissues. **j**, **k** Immunofluorescence images and quantification of CSPGs intensity in the mouse tissues. Quantitative data were presented as means ± SD (*n* = 3, unless otherwise noted). *, *p* < 0.05; **, *p* < 0.01; ***, *p* < 0.001; ****, *p* < 0.0001. *p* values were calculated by two-tailed unpaired Student's t-test for comparisons between two groups, and by one-way ANOVA for multiple comparisons. Scale bars represent 100 μm

To investigate if excessive glutamate would promote MAO-B expression in astrocytes, we treated astrocytes with glutamate and found an increase in EAAT1 (2.5-fold) and MAO-B expression (1.9-fold) (Fig. 3b-d). There was a decrease in MAO-B expression when blocking the glutamate transport activity in astrocytes using glutamate transporter inhibitor (TBOA) (Fig. 3b, d). As H_2_O_2_ is a by-product generated upon activity of MAO-B enzyme [14, 15], we investigated if glutamate treatment also promotes H_2_O_2_ in astrocytes. Upon glutamate treatment, we stained astrocytes with ROS indicator and found an increase in ROS intensity in the glutamate-treated astrocytes compared to the non-treated astrocytes (Supplementary Fig. 7). In addition, we found a significant GFAP increase (3.5-fold) upon treatment with glutamate compared to non-treated astrocytes, which were significantly reduced by TBOA and KDS2010 (Fig. 3e, f). Interestingly, we also found a significant increase in CSPGs (1.9-fold) from glutamate-treated astrocytes, which was decreased with TBOA and KDS2010 treatment (Fig. 3g). These results suggested that MAO-B upregulation mediated by glutamate was a potential mediator for reactive astrogliosis and astrocytic scar formation.

To confirm the role of MAO-B for astrocytic scar in vivo, we treated the nude mice with MAO-B inhibitor (KDS2010, 10 mg/kg/day) for 14 days by oral gavage, starting from day 7 after GFP-labeled U87 MG injection. We found a significant decrease in GFAP (Fig. 3h, i) and CSPGs (Fig. 3j, k) expression in the tumor periphery region, which did not overlap with the GBM region (GFP-labeled U87 MG), of KDS2010-treated mice. This result suggested that MAO-B was a potential mediator for glial scar formation, which was consistent with findings from our *in vitro* model. However, it should be noted that there was an overlap between strong GFAP signal and GFP-labeled GBM (white arrowhead in Fig. 3h) at the tumor boundary, suggesting that GFAP signals in this area might originate from both astrocytes and peripheral GBM cells. The proposed mechanism for the induction of reactive astrocytes and scar formation mediated by glutamate–MAO-B activity was summarized in Fig. 3a. Glutamate transported through EAAT1 transporter increased MAO-B enzyme expression in astrocytes, which consequently generated H_2_O_2_ as a by-product of MAO-B. The increase of MAO-B induced reactive astrogliosis, shown by an increase in GFAP expression. In addition, CSPGs were deposited as an extracellular matrix released from reactive astrocytes. Taken together, our data suggested that glutamate–MAO-B activity was a critical modulator for the reactive astrogliosis and scar formation.

### Limiting GBM growth by the astrocytic scar

**Fig. 4:**
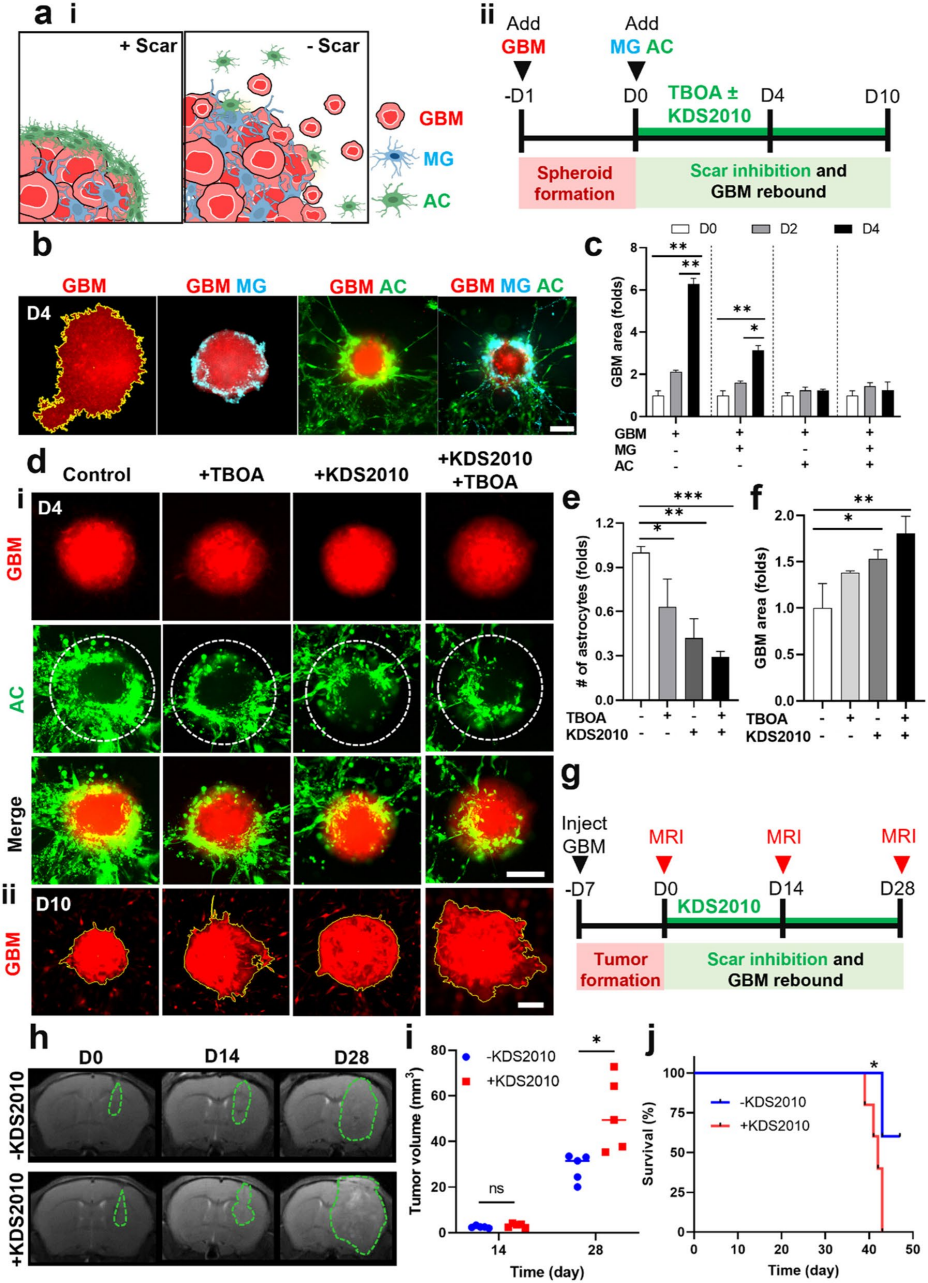
Limiting GBM growth by the astrocytic scar. **a** i) Schematic illustrating the role of astrocytic scar in limiting GBM growth (‘ + Scar’), and GBM regrowth upon scar inhibition (‘- Scar’). ii) Timeline for the assembloid construction assessing GBM rebound after scar inhibition with TBOA and KDS2010. **b**, **c** Limiting GBM growth by the glial scar barrier. **d** Inhibition of the astrocytic scar promoting GBM regrowth. i) Fluorescent images of glial scar-GBM assembloid at day 4 (D4) showing the inhibition of astrocytic scar formation by TBOA and KDS2010. ii) Fluorescent images of glial scar-GBM assembloid at day 10 (D10) with TBOA and KDS2010 treatment, showing GBM regrowth after scar inhibition. **e** Quantification of scar-forming astrocytes at D4 within the scar-GBM circles (white dashed circles in **d**-**i**) from the middle plane of confocal images. **f** Quantification of the GBM size at D10, showing the GBM rebound after scar inhibition. **g** Experimental protocol for assessing *in vivo* GBM regrowth with scar inhibition. **h**-**i** MRI images and quantification of tumor volume with KDS2010 treatment. **j** Mouse survival rate with KDS2010 treatment. Data were presented as means ± SD (*n* = 3, unless otherwise noted). *, *p* < 0.05; **, *p* < 0.01; ***, *p* < 0.001; ****, *p* < 0.0001. *p* values were calculated by one-way ANOVA for multiple comparisons. Two-way ANOVA was used to compare between groups with two independent variables. Differences in survival rate was tested by log-rank (Mantel-Cox) test. Scale bars represent 200 μm. GBM, glioblastoma; MG, microglia; AC, astrocytes

To examine whether reactive astrogliosis and scar formation could limit the growth of GBMs, we treated the glial scar-GBM *in vitro* model with TBOA and KDS2010 to inhibit astrocytic scar and examined the GBM growth (Fig. 4a-i). Timeline for the model construction assessing GBM growth with scar inhibition was summarized in Fig. 4a-ii. Over a 4-day cultural period, GBM spheroids continued growing in single culture model (6.3-fold) and co-cultured with microglia (GBM MG) (3.1-fold) (Fig. 4b, c). However, there were no significant changes in the area of GBM spheroids cocultured with astrocytes (GBM AC) and microglia/astrocytes (GBM MG AC) (Fig. 4b, c), indicating that astrocytes limit GBM growth. Next, we investigated if the inhibition of glial scar formation would allow the GBM regrowth. Since glutamate–MAO-B activity was demonstrated to be a mediator for astrogliosis and scar formation (Fig. 3e-k), we investigated whether the astrocytic scar targeting GBM-microglia assembloid would be inhibited upon treatment with glutamate transporter inhibitor (100 μM TBOA) and/or MAO-B inhibitor (1 μM KDS2010). We observed a significant decrease of scar-forming astrocytes upon treatment with TBOA and KDS2010 for 4 days (Fig. 4d-i, e). TBOA/KDS2010 combination treatment could reduce up to 71% of scar-forming astrocytes, quantified by counting the number of astrocytes in the middle plane of confocal images (Fig. 4e). In addition, we observed an increase in GBM area (1.8-fold) at day 10 when inhibiting the astrocytic scar with TBOA and KDS2010 (Fig. 4d-ii, f). We replicated the scar attenuation with TBOA/KDS2010 using serum-free media and found compatible results with 10% FBS media (Supplementary Fig. 2b-d). These results indicated that the glial scar barrier could limit GBM growth, while inhibition of glutamate–MAO-B activity attenuated the astrocytic scar which allowed GBM regrowth.

Next, we investigated if tumor growth could be limited by the astrocytic scar barrier *in vivo*. After 1-week implantation of tumor cells, the mice with visible brain tumors were randomly divided into two groups: one group treated with KDS2010 (10 mg/kg/day) by oral gavage for 28 days, and the other group not received treatment (Fig. 4g). On day 28, the MRI images showed a larger tumor size in the KDS2010-treated mice (+ KDS2010) compared to that of non-treated mice (-KDS2010) (Fig. 4h, i). Mice receiving KDS2010 (+ KDS2010) had significantly shorter survival than the control group (-KDS2010) (Fig. 4j), indicating that astrocytic scar limits GBM growth and extends survival rate *in vivo*. In sum, our results indicated that the astrocytic scarring phenomenon played a crucial role in restricting tumor growth, which could be modulated via the glutamate–MAO-B activity.

### Increased drug infiltration to GBM by inhibiting astrocytic scar formation

**Fig. 5:**
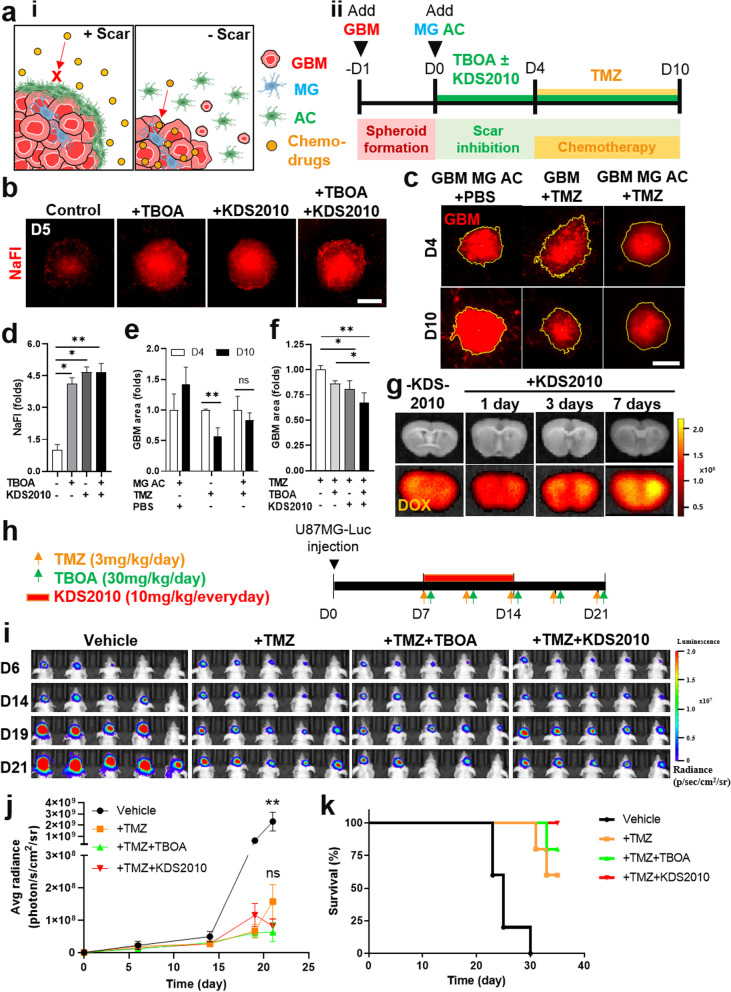
Increased drug infiltration to GBM by inhibiting astrocytic scar formation. **a** i) Schematic illustrating the limited chemo-drug infiltration to GBM region due to the astrocytic scar barrier. ii) Timeline for assessing drug infiltration to GBM with scar inhibition *in* *vitro*. **b**, **d** Fluorescent images and quantitative results for the intensity of fluorescein sodium salt (NaFI, MW 376 Da), representing the increased infiltration of small molecular to GBM region induced by the scar inhibition. **c**, **e** Fluorescent images and quantitative results of GBM spheroid area, representing decreased sensitivity to TMZ treatment by the glial scar. Without the glial scar, GBM spheroids reduced their area after TMZ treatment (D10) compared to that of before treatment (D4) (GBM + TMZ). With the glial scar, no significant reduction in GBM spheroid area were observed after treatment with TMZ (GBM MG AC + TMZ). **f** Increased sensitivity to TMZ with scar inhibition by TBOA and KDS2010 *in* *vitro*. **g** Ex vivo images representing the increased DOX penetration after scar inhibition (*n* = 2). **h** Experimental protocol for assessing drug sensitivity with scar inhibition *in vivo*. **i**-**j** Bioluminescence images and quantification of GBM tumor upon TMZ treatment with scar inhibition by TBOA and KDS2010 (*n* = 5). **k** Survival rate of mice (*n* = 5. Data were presented as means ± SD (*n* = 3, unless otherwise noted), except data in 5j which was presented in means ± SEM. *, *p* < 0.05; **, *p* < 0.01; ****, *p* < 0.0001. *p* values were calculated by two-tailed unpaired Student’s t-test for comparisons between two groups, and by one-way ANOVA for multiple comparisons. Differences in survival rate was tested by log-rank (Mantel-Cox) test. DOX, Doxorubicin; TMZ, Temozolomide. Scale bars represent 200 μm

Since the astrocytic scar limited GBM growth (Fig. 4b-f and h, i), we further investigated if the scar barrier also limited drug infiltration into GBM (Fig. 5a). We used fluorescein sodium salt (NaFI, MW 376 Da), a non-toxic fluorescent tracer, which has compatible molecular weight to TMZ to investigate drug diffusion through the scar barrier. Upon the glial scar formation (day 4), we treated the assembloid with astrocytic scar model with 0.5 μg mL^−1^ NaFI for 24 h. We observed a significant increase of NaFI intensity in the GBM region when inhibiting the astrocytic scar with TBOA (4.1-fold), KDS2010 (4.7-fold), and combined TBOA and KDS2010 treatment (4.7-fold) (Fig. 5b, d). Next, we single cultured GBM spheroids in Matrigel and co-cultured GBM spheroids with glial cells (GBM MG AC) (Fig. 5c, e). Upon model construction (day 4), we treated them with 500 μM TMZ, an FDA-approved small molecular drug for GBM patients [27], and used PBS in control treatment (Fig. 5c, e). After the treatment (day 10), there was no significant decrease in the area of GBM with glial scar (GBM MG AC + TMZ), while GBM without the glial scar significantly decreased in area (GBM + TMZ) (Fig. 5c, e). It should be noted that IFN-γ and IL-10 were not significantly detected in our model (Supplementary Fig. 8). These results suggested that the decreased drug diffusion across the scar barrier is a potential reason for reduced drug efficacy in GBM. Next, we explored if we could increase drug sensitivity by inhibiting the astrocytic scar. As TBOA and KDS2010 attenuated the astrocytic scar (Fig. 4d-i, e), we treated the assembloid with astrocytic scar model with 100 μM TBOA and/or 1 μM KDS2010; and used TMZ (500 μM) as a chemotherapy drug (Fig. 5a-ii, f). Upon drug treatment (day 10), the area of GBM treated with TMZ + TBOA and TMZ + KDS2010 reduced more significant than GBM treated with TMZ alone (Fig. 5f). We replicated this finding using serum-free media and found compatible results (Supplementary Fig. 2e-f). This result represented an increased sensitivity to TMZ by inhibiting the astrocytic scar via glutamate–MAO-B activity *in* *vitro*.

Next, we explored drug infiltration to GBM region and drug sensitivity upon scar inhibition *in vivo*. One week after the implantation of tumor cells, we inhibited the astrocytic scar formation by oral administration of KDS2010 with different treatment duration (1 day, 3 days, and 7 days). Then, DOX (MW 544 Da) was intravenously administered to the tumor-bearing mice and ex vivo imaging was performed by using IVIS. We observed higher fluorescence signals of DOX in 7-day KDS2010-treated mice than that of 1-day and 3-day treated mice, while mice without treatment (-KDS2010) showed limited DOX intensity (Fig. 5g). This result suggested that inhibition of the astrocytic scar promoted small molecular drug penetration. To assess real-time tumor growth, U87 MG-Luciferase stable cells were transplanted into nude mice. Seven days after transplantation, we treated the tumor-bearing mice with TMZ (3 mg/kg/day), TBOA (30 mg/kg/day) at 3-day intervals, and KDS2010 (10 mg/kg/day) daily for a week (Fig. 5h). GFAP immunostaining on mouse brain showed that astrocytic scar was attenuated with TBOA and KDS2010 treatment (Supplementary Fig. 9), however, bioluminescence image analyses showed that there were no significant differences in tumor size between groups treated with + TMZ + TBOA and + TMZ + KDS2010, compared to the group treated with TMZ alone (Fig. 5i, j). The + TMZ + TBOA and + TMZ + KDS2010 treatment conditions did not improve the survival rate of mice as a result (Fig. 5k). Taken together, these results validated that the astrocytic scar barrier limited drug infiltration to GBM region, which could be increased by inhibition of the glutamate–MAO-B activity.

### Astrocytic scar limiting human brain tumor growth by glutamate–MAO-B activity

**Fig. 6:**
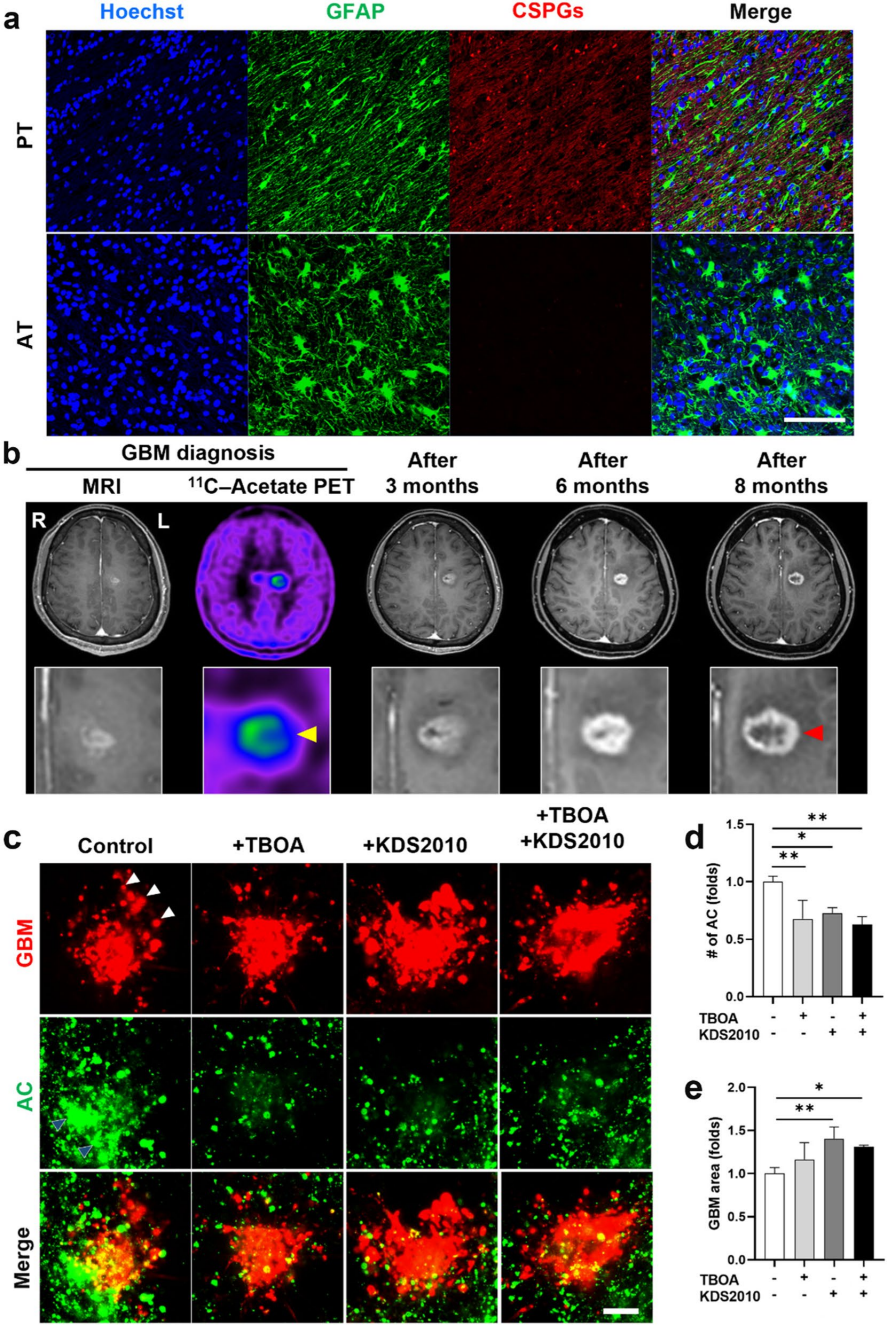
Astrocytic scar limiting human brain tumor growth via glutamate–MAO-B activity. **a** Presence of astrocytic scar formation in human GBM brain tissue assessed by immunostaining. GFAP^+^ cells adjacent to GBM tumor (PT region) transformed to elongated shape and highly expressed CSPGs, while GFAP^+^ cells far away from GBM tumor (AT region) maintained their star shape with no significant CSPGs expression. **b** A clinical case showing astrocytic scar limiting human brain tumor growth assessed by MRI and ^11^C–Acetate PET. ^11^C–Acetate PET image showed high ^11^C–Acetate uptake in the right (R) axis and low ^11^C–Acetate uptake in the left (L) axis (yellow arrowhead), representing regions with high and low density of reactive astrocytes, respectively. MRI images at 3, 6, and 8 months showed ring-enhancing lesions with higher contrast in the left axis (red arrowhead), suggesting the tumor was outgrowing towards left side which had low density of reactive astrocytes. **c** Inhibition of glutamate–MAO-B attenuating astrocytic scar while promoting tumor growth in GBM patient-derived assembloids. White arrowheads indicates the invasion of GBM cells to region with low density of astrocytes. **d** Quantitative data for the number of scar-forming astrocytes in the GBM patient-derived assembloid. **e** Quantitative data for GBM area in the GBM patient-derived assembloid. *, *p* < 0.05; **, *p* < 0.01. *p* values were calculated by one-way ANOVA for multiple comparison. Scale bars represent 100 μm. PT, peri-tumor; AT, away from tumor; MRI, magnetic resonance imaging; PET, positron emission tomography

To assess the clinical importance of astrocytic scar barrier in limiting human brain tumor growth, we first validated the presence of astrocytic scar formation in human brain tumor by immunostaining the GBM patient brain tissue with GFAP and CSPGs. We observed GFAP^+^ cells with elongated shape in the region adjacent to GBM tumor mass (labeled as PT region), while GFAP^+^ cells far away from GBM exhibited star shapes (labeled as AT region) (Fig. 6a), which was compatible to observation in our assembloid and mouse xenograft model. CSPGs were highly expressed in the PT region, while no significant CSPGs was observed in the AT region (Fig. 6a). We also immunostained the human GBM tissue with IBA1 and GFAP to assess the distribution of microglia/astrocytes in accordance with GBM tumor. Interestingly, we found IBA1^+^ cells presented both inside and outside GBM tumor, while GFAP^+^ cells mainly accumulated surrounding tumor mass (Supplementary Fig. 10a). It should be noted that GFAP signals were also expressed at the intratumor regions where GBM cells were located (Supplementary Fig. 10b). Then, we investigated the ability of astrocytic scar in limiting tumor growth in the human brain. Since ^11^C–Acetate was highly uptake by reactive astrocytes in the glial scar barrier, we used ^11^C–Acetate positron emission tomography (PET) imaging technique to observe glial scar regions in GBM patient brain [24, 28, 29]. We detected high ^11^C–Acetate uptake in the right (R) axis and low ^11^C–Acetate uptake in the left (L) axis in the brain of a GBM patient at the time of tumor diagnosis, suggesting high and low density of reactive astrocytes in the right and left sides of brain tumor, respectively (Fig. 6b). The MRI images taken at 3, 6, and 8 months after diagnosis showed ring-enhancing lesions with higher contrast in the left axis compared to that of right axis, suggesting that brain tumor grew faster towards the left side which had low density of reactive astrocytes (Fig. 6b). Next, we investigated if the astrocytic scar formation in human brain tumor was mediated by glutamate–MAO-B activity as observed in the assembloids and mouse xenograft models. We formed the GBM patient-derived assembloid by triculturing patient-derived GBM spheres and immortalized human microglia/astrocytes. Over 8 days, astrocytes migrated towards GBM patient spheres (Fig. 6c), while number of migrated astrocytes were significantly reduced with TBOA, KDS2010, or TBOA/KDS2010 treatment (Fig. 6c, d). We observed patient-derived GBM cells invading regions with low density of reactive astrocytes (white arrowheads) (Fig. 6c). In addition, there were an increase in GBM area of the assembloid treated with TBOA, KDS2010, or TBOA/KDS2010 (Fig. 6e). Taken together, these results suggested that astrocytic scar was able to limit human brain tumor growth via glutamate–MAO-B activity.

## Discussion

The glial scar formation has been generally observed after an injury or an inflammation in the central nervous system. However, the underlying mechanism for the glial scar formation remains elusive. Here, we constructed for the first time an *in* *vitro* human astrocytic scar in a GBM-microglia assembloid mimicking the glial scar formation responding to GBM tumor microenvironment. We discovered that GBM and microglia interplay augmented glutamate level in the GBM microenvironment and the glutamate–MAO-B activity played an essential role for the astrocytic scar formation. We found that the glial scar served as a physical barrier limiting GBM growth and attenuation of the astrocytic scar promoted drug infiltration to GBM region. This finding was consistently recapitulated in a mouse tumor xenograft model.

First, we constructed physiologically relevant astrocytic scar in an assembloid model mimicking the glial scar formation in human GBM, characterized by the glial cell distribution, morphology, and reactivity. We employed spheroids as 3D cell culture model to recapitulate the GBM tumor mass as spheroids can mimic the structure and drug infiltration properties of tumor [30]. The GBM spheroids were cultured in 3D Matrigel containing homogenous mixture of astrocytes and microglia. Through this model, we observed that microglia gradually accumulated at the interface of GBM and infiltrated into the GBM spheroid, forming the GBM-microglia assembloid. This phenomenon was similar to the observation on our *in vivo* tumor xenograft model and GBM patient tissue. Supporting this finding is an observation on the GBM-grafted mice that microglia actively wrapped around GBM tumor and intermingled with GBM cells inside the tumor [31]. Astrocytes also accumulated at the interface of GBM-microglia assembloid, forming the astrocytic scar barrier. The scar-forming astrocytes became reactive and transformed their morphology to elongated shape, which was in line with a study by Wanner et al*.* who reported the elongated morphology of astrocytes in the mature scar adjacent to lesions [26]. Taken together, these data indicated that our model was relevant for the mechanism study of reactive gliosis and scar formation in GBM.

We explored the mechanism of glial scar formation responding to GBM. We found the GBM-microglia interplay significantly increased glutamate level in the tumor microenvironment. Neutralization of cytokines (GM-CSF, IL-6, and G-CSF) decreased glutamate released from microglia, suggesting the role of these cytokines mediating microglial glutamate in the interplay. Then, we investigated how glutamate induced reactive astrogliosis and astrocytic scar formation. Our previous study revealed that MAO-B was a potential mediator involving in the reactivity of astrocytes [16]. In this study, we disclosed that excessive glutamate originating from GBM-microglia assembloid increased MAO-B expression in astrocytes, leading to an increase in H_2_O_2_ as a by-product of MAO-B. Our study confirmed the induction of reactive astrogliosis (GFAP) and the release of extracellular matrix (CSPGs) from reactive astrocytes upon MAO-B upregulation. In this regard, we suggested that glutamate–MAO-B activity is a critical modulator for the astrogliosis and scar formation.

Finally, we found dual roles of the glial scar for inhibiting GBM growth and limiting drug infiltration. Limiting GBM growth could be due to the barrier-like structure of the astrocytic scar as we observed an increased GBM regrowth when we decreased the number of scar-forming astrocytes in assembloid and mouse xenograft models. We also observed the tumor outgrowth towards region having low density of reactive astrocytes in the brain of a GBM patient. Although we observed this phenomenon in a single clinical case since most GBM patients undergo tumor resection surgery shortly after diagnosis, this observation lay a strong foundation for future clinical study investigating the role of glial scar for restricting tumor cell expansion. In addition, we found a resistance to TMZ in our model. Although drug resistance induced by the glial scar is contributed by many factors such as IL-10 and IFN-γ from reactive astrocytes [32], through our model, we suggested that decreased drug infiltration to GBM region due to glial scar barrier was a potential reason leading to drug resistance. This view is further supported by the *in* *vitro* evidence showing an increased sensitivity to TMZ when inhibiting the astrocytic scar formation. Although we could recapitulate the increased drug infiltration into mouse brain with scar inhibition, we were not able to increase drug sensitivity *in* *vivo* and we could not prolong mice survival using chemotherapy combined with scar inhibition. This observation could be explained by the complex cellular crosstalk of other cell types in the *in* *vivo* model leading to drug resistance [33–35]. In the future study, we plan to explore a sweet spot of scar inhibition level at which the scar could limit GBM growth while minimizing drug resistance. Taken together, we suggested that the astrocytic scar was a potential physical barrier that could be modulated to limit GBM growth and promote drug infiltration to GBM.

In the current study, we developed a human brain tumor microenvironment model mimicking the glial scar formation responding to GBM. To the best of our knowledge, our model is the first *in* *vitro* model mimicking the glial scar formation. By using our model, we could separately investigate the role of microglia and astrocytes in the scar formation, which is difficult to explore on animal models and human samples. Through this model, we also discovered a beneficial aspect of GBM-glial cell crosstalk, adding a new dimension to the existing theory of tumor microenvironment. For decades, the GBM-glia crosstalk has been considered a detrimental phenomenon, which promotes tumor progression and chemotherapy resistance [36]. For example, a spheroid model formed by homogenously mixing GBM and astrocytes (ratio 1:1) reported a decreased drug sensitivity of GBM caused by astrocytes [37]. However, astrocytes account for only 1% inside GBM tumor, while most of them are located outside the tumor mass [38]. In our model, we cultured GBM spheroids surrounded by glial cells in 3D Matrigel. By doing this, we could observe the glial scarring phenomenon and discovered the beneficial role of GBM-glia crosstalk which could not be observed in other coculture models. In this regard, we believe our model is relevant for studying the role of individual glial cell type forming the scar and the role of glial scar for tumor. However, there are several contentions and limitations of our study that we should take into consideration. Glial scar barrier exhibited both beneficial effect by restricting GBM growth and detrimental effect by limiting drug infiltration to tumor region. Although attenuation of glial scar increased drug infiltration to GBM region and subsequently increased drug sensitivity in our assembloid, generalization of this finding to increase drug sensitivity in *in* *vivo* models should take into consideration other tumor microenvironment factors contributing to drug resistance [39]; and should clearly identify a sweet spot of scar attenuation level to prevent tumor escaping through the loosen glial scar barrier. In addition, although our glial scar-GBM assembloid composed major cell types involving in glial scar formation, the GBM microenvironment was highly heterogeneous composing of other innate immune cells such as perivascular/peripheral macrophages and monocytes [40]. Previous studies reported glutamate released from macrophages/monocytes during inflammation [41, 42], indicating their potential contribution to the astrocytic scar formation. Future study might consider including these cell types in the assembloid to investigate their roles for the astrocytic scar formation.

## Conclusions

In conclusion, we presented here a 3D human astrocytic scar targeting GBM-microglia assembloid, mimicking the glial scar formation in GBM. Through our model, we found that glutamate–MAO-B was a critical modulator for the astrocytic scar formation. We have clarified that the glial scar was a potential physical barrier for limiting GBM growth. Attenuation of the scar increased drug infiltration to GBM region, but this strategy might trigger tumor cells escaping through the loosen glial scar barrier. We believe that our study will contribute to a greater understanding of the scar formation mechanism and provide a solid foundation for future therapeutic strategy overcoming glial scar barrier. We envision that our human astrocytic scar model could serve as a reliable platform for studying drug delivery across the scar barrier.

## Supplementary Information

**Additional file 1.** **FigureS1. **Schematicfor the in vitro GBM-glial scarmodel construction. **Figure S2.** Glial scar-GBMassembloid model construction using serum-free media. **Figure S3.**Astrocytic reactivity. **Figure S4.**Scar-GBM circle. **Figure S5.** Intratumor, peritumor, and away from tumorregion. **Figure S6.** GFAP expressionat the IT region. **Figure S7.** H_2_O_2_expression with glutamate treatment. **Figure S8. **IL-10 and IFN-γ expression in the assembloid model. **FigureS9.** GFAP expression in mouse brain tissue. **Figure S10.** Distribution of GFAP^+^ and Iba1^+^cells in human GBM tissue. **Table S1. **Summary of statistical analyses **TableS2. **Summary of outcome measures.

**Additional file 2.** Checklistfor the ARRIVE guideline.

## Data Availability

The datasets used and/or analysed during the current study are available from the corresponding author on reasonable request.

## Abbreviations

3D: Three-dimensional
ADP: 5’-Diphosphate
AT: Away from tumor
CSPGs: Chondroitin sulfate proteoglycans
DOX: Doxorubicin
EAATs: Excitatory amino acid transporters
FBS: Fetal bovine serum
GBM: Glioblastoma multiforme
G-CSF: Granulocyte colony-stimulating factor
GFAP: Glial fibrillary acidic protein
GFP: Green fluorescent protein
GM-CSF: Granulocyte-macrophage colony-stimulating factor
IL-6: Interleukin 6
IT: Intratumor
IVIS: In vivo imaging system
L/W: Length/width
L-GLDH: Glutamic dehydrogenase
MAO-B: Monoamine oxidase B
MRI: Magnetic resonance imaging
NAD: Adenine dinucleotide
PBS: Phosphate-buffered saline
PFA: Paraformaldehyde
PT: Peri-tumor
ROS: Reactive oxygen species
TMZ: Temozolomide
