# Supplementary material for: Astrocytic scar restricting glioblastoma via glutamate–MAO-B activity in glioblastoma-microglia assembloid
Source: Biomater Res. 2023 Jul 19;27:71. doi: 10.1186/s40824-023-00408-4 (PMC10355029; doi:10.1186/s40824-023-00408-4)
Supplement: Supplementary file 1 — Additional file 1. FigureS1. Schematicfor the in vitro GBM-glial scarmodel construction. Figure S2. Glial scar-GBMassembloid model construction using serum-free media. Figure S3.Astrocytic reactivity. Figure S4.Scar-GBM circle. Figure S5. Intratumor, peritumor, and away from tumorregion. Figure S6. GFAP expressionat the IT region. Figure S7. H2O2expression with glutamate treatment. Figure S8. IL-10 and IFN-γ expression in the assembloid model. FigureS9. GFAP expression in mouse brain tissue. Figure S10. Distribution of GFAP+ and Iba1+cells in human GBM tissue. Table S1. Summary of statistical analyses TableS2. Summary of outcome measures. [file 40824_2023_408_MOESM1_ESM.docx]

**Supplementary data**

**Astrocytic Scar Restricting Glioblastoma via Glutamate–MAO-B Activity**

**In Glioblastoma-Microglia Assembloid**

Yen N. Diep^1-3,11^, Hee Jung Park^4,11^, Joon-Ho Kwon^5,6,11^, Minh Tran^1-3^, Hae Young Ko^4^, Hanhee Jo^4^, Jisu Kim^4^, Jee-In Chung^4^, Tai Young Kim^5^, Dongwoo Kim^4^, Jong Hee Chang^7^, You Jung Kang^1-2^, C. Justin Lee^5,6,8-10^*, Mijin Yun^4^* and Hansang Cho^1-3^*

^1^Institute of Quantum Biophysics, Sungkyunkwan University, Suwon, 16419, Republic of Korea

^2^Department of Biophysics, Sungkyunkwan University, Suwon, 16419, Republic of Korea

^3^Department of Intelligent Precision Healthcare Convergence, Sungkyunkwan University, Suwon, 16419, Republic of Korea

^4^Department of Nuclear Medicine, Yonsei University College of Medicine, Seoul, 03722, Republic of Korea

^5^Center for Cognition and Sociality, Institute for Basic Science, Daejeon, 34126, Republic of Korea

^6^Department of Biomedical Engineering, Ulsan National Institute of Science & Technology, Ulsan, 44919, Republic of Korea

^7^Department of Neurosurgery, Severance Hospital, Seoul, 120-752, Republic of Korea

^8^Korea University-Korea Institute of Science and Technology, Graduate School of Convergence Technology, Korea University, Seoul, 136-701, Republic of Korea

^9^IBS School, University of Science and Technology, Daejeon, 34113, Republic of Korea

^10^Institute for Life Sciences, Institute for Basic Science

^11^These authors contributed equally.

*Corresponding to [h.cho@g.skku.edu](mailto:h.cho@g.skku.edu) (Hansang Cho); [YUNMIJIN@yuhs.ac](mailto:YUNMIJINyunmijin@yuhs.ac) (Mijin Yun); [cjl@ibs.re.kr](mailto:cjl@ibs.re.kr) (C. Justin Lee) at 2066 Seobu-ro, Suwon-si, Gyeonggi-do, Republic of Korea.

**
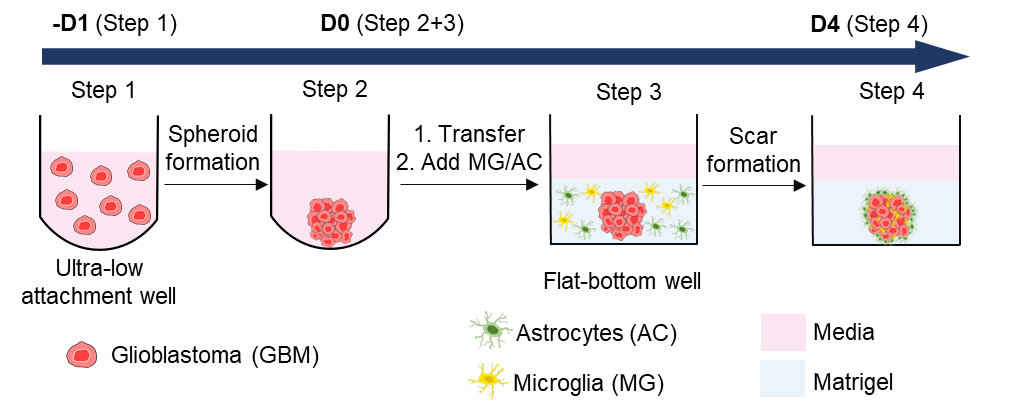
**

**Supplementary Figure 1.** **Schematic for the *in vitro* GBM-glial scar (GBM assembloid) model construction.**

GBM spheroids were formed using the ultra-low attachment well plate. The spheroids were transferred to flat-bottom wells and co-cultured with microglia/astrocytes mixed in 3D Matrigel. After polymerization of Matrigel, media was added to the wells. The glial scar formation were observed for 4 days.


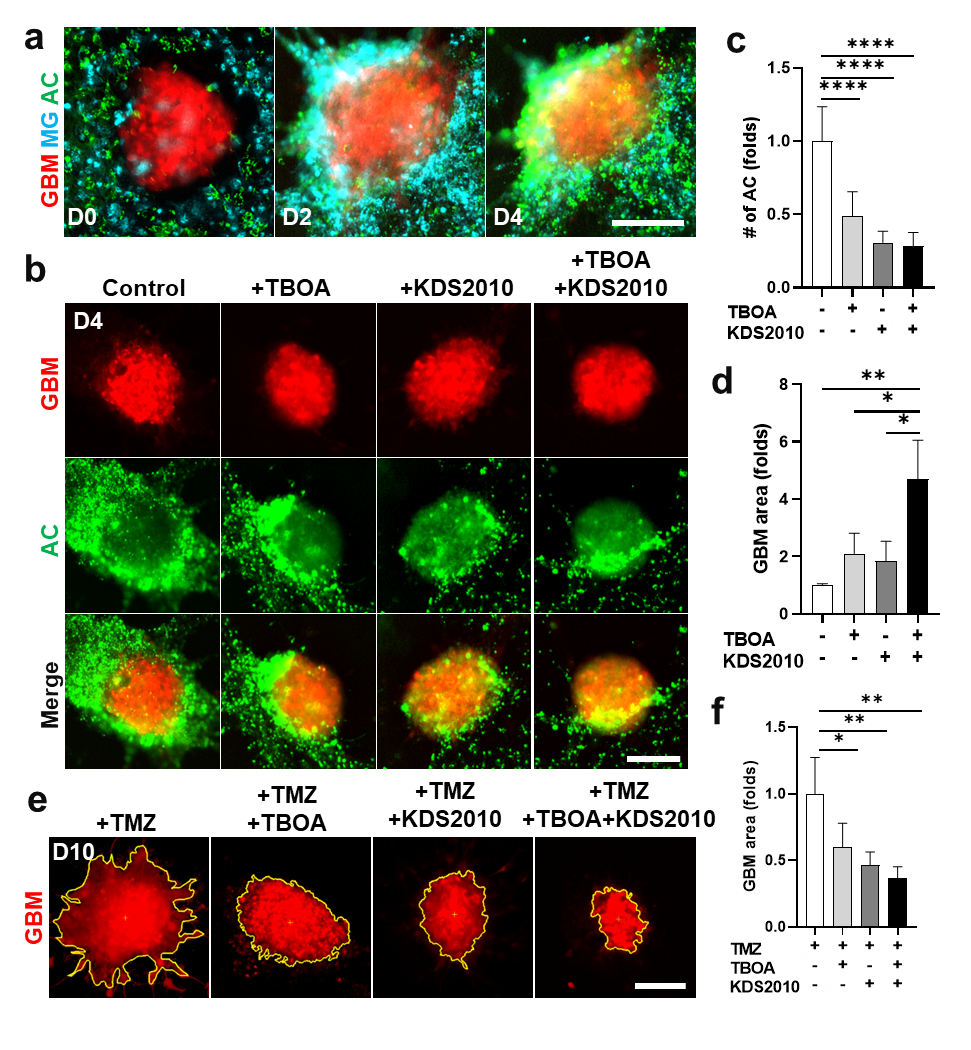
**Supplementary Figure 2. Glial scar-GBM assembloid model construction using serum-free media.**

a) Time-lapse images of the glial scar formation. b) Inhibition of astrocytic scar formation using TBOA and KDS2010. c) Quantification of scar-forming astrocytes at D4, counted from the middle plane of confocal images (n = 6). d) GBM area at D10, showing the GBM rebound upon scar inhibition. e-f) Fluorescent images and quantitative results of GBM area showing the increased sensitivity to TMZ with scar inhibition by TBOA and KDS2010 (n = 4). Data were presented as means ± SD (n = 3, unless otherwise noted). *, *p* < 0.05; **, *p* < 0.01; ****, *p* < 0.0001. *p* values were calculated by one-way ANOVA for multiple comparisons. TMZ, Temozolomide. Scale bars represent 200 µm.

**
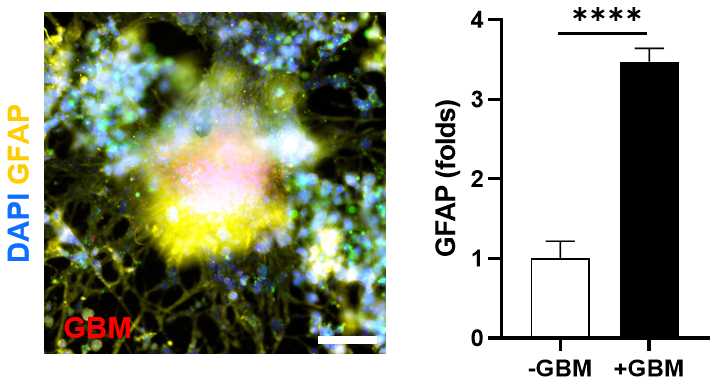
**

**Supplementary Figure 3. Astrocytic reactivity.**

Reactivity of astrocytes in the *in vitro* glial scar-GBM assembloid assessed by immunostaining with GFAP. Scale bar represents 200 µm.


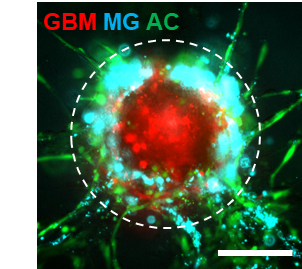


**Supplementary Figure 4. Scar-GBM circle.**

The 500-µm diameter circle (white dashed circle) with the center point same as GBM spheroids were used for quantification of scar-forming glial cells. Scale bar represents 200 µm.


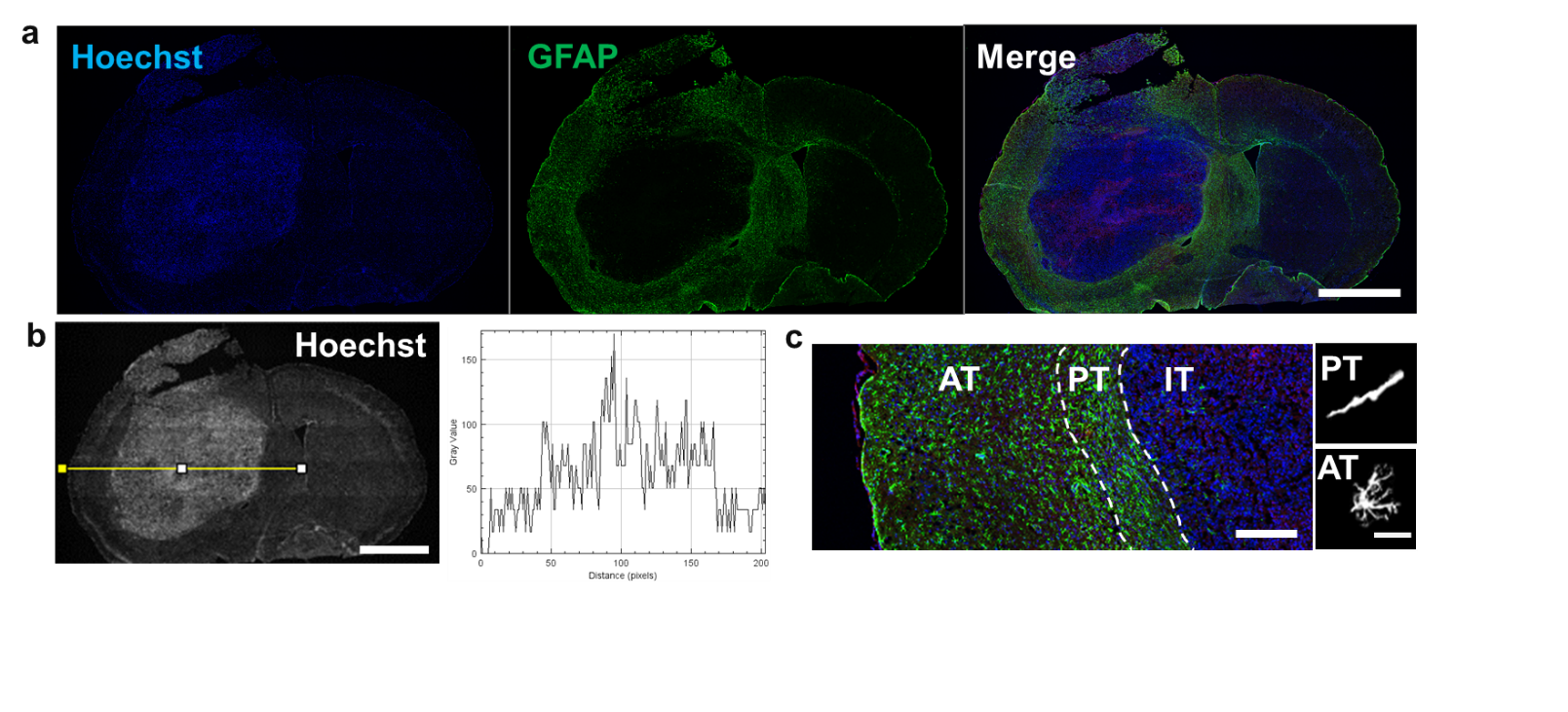
*Intratumor (IT), peritumor (PT), and away from tumor (AT) region in vivo*: As we observed a higher cellular density in GBM tumor mass compared to surrounding area, we determined the region with higher cellular density as tumor margin, called *intratumor (IT) region* (Supplementary Figure 5a,b). In addition, we observed a transformation in morphology of astrocytes to elongated shape, located within 200 µm from the tumor margin (Supplementary Figure 5c). Therefore, we determined the region from tumor margin to tumor margin+200µm as *peritumor (PT) region* (Supplementary Figure 5c). The region from tumor margin+ >200µm was called *away from tumor (AT) region* (Supplementary Figure 5c).

**Supplementary** **Figure 5**. **Intratumor (IT), peritumor (PT), and away from tumor (AT) region.**

a) Immunostaining images showing the location of GBM tumor surrounding by astrocytes (GFAP^+^). Scale bar represents 1 mm. b) Greyscale image and quantification showing higher cellular density in GBM tumor mass. Scale bar represents 1 mm. c) Morphology of astrocytes at the PT and AT region. Scale bar represents 200 µm. Scale bar in the zoom-in image represent 25 µm.


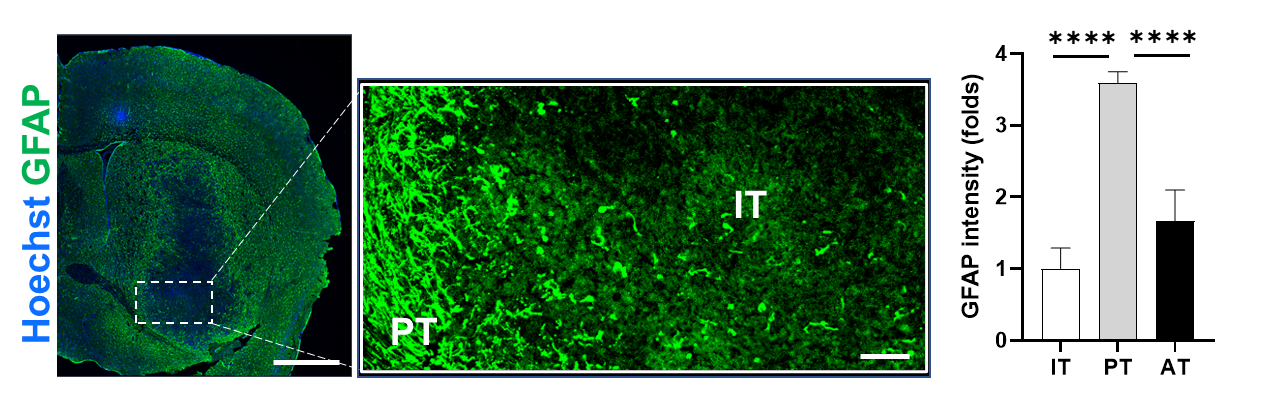


**Supplementary Figure 6. GFAP expression at the IT region.**

Positive GFAP signals at the IT region, but their intensity was significantly lower than that of PT and AT regions. Data were presented as means ± SD. ****, *p* < 0.0001. *p* values were calculated by one-way ANOVA for multiple comparisons.


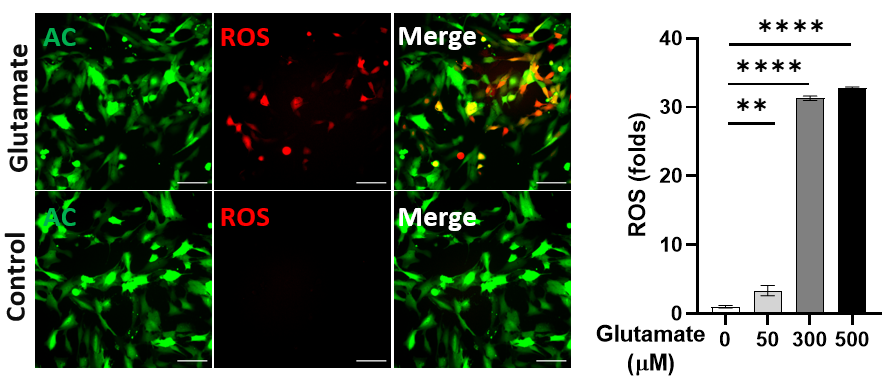


**Supplementary** **Figure 7. H_2_O_2_ expression with glutamate treatment.**

Increased H_2_O_2_ in astrocytes with glutamate treatment assessed by ROS indicator.


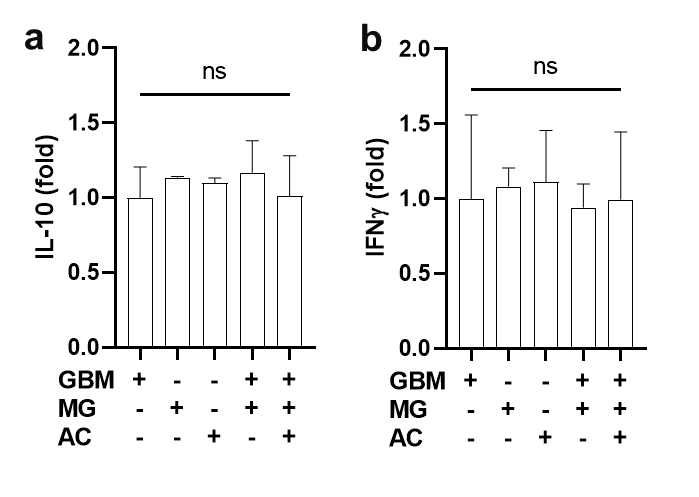
**Supplementary Figure 8. IL-10 and IFN-γ expression in the assembloid model.**

No significant expression of IL-10 and IFN-γ assessed by human cytokine assay.


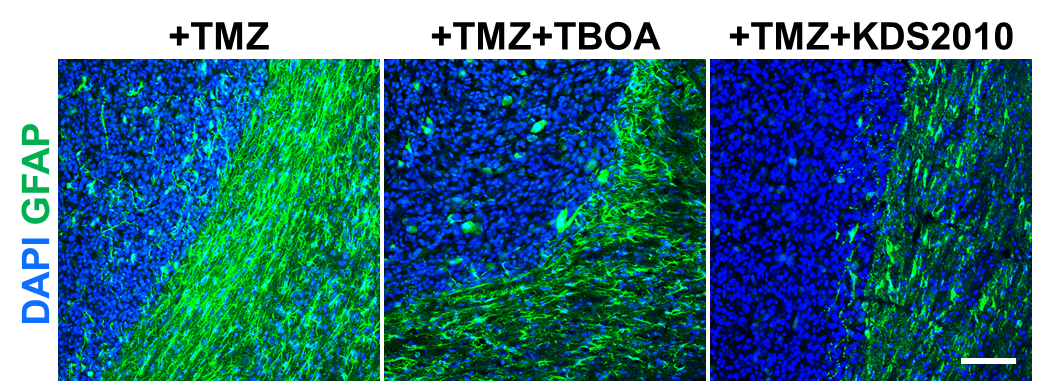


**Supplementary Figure 9.** **GFAP expression in mouse brain tissue.**

Attenuation of astrocytic scar with TBOA and KDS2010 treatment assessed by immunostaining with GFAP. Scale bar 100 µm.

**
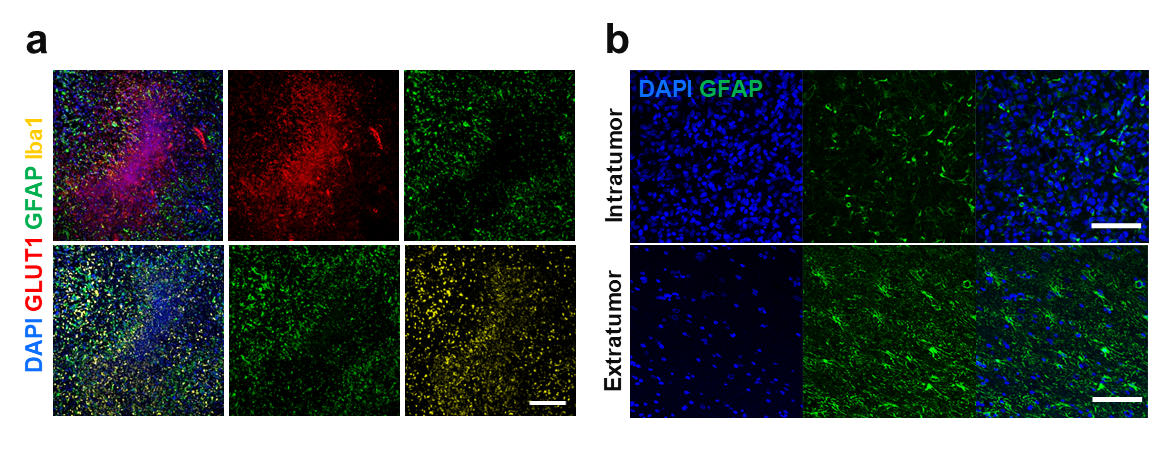
Supplementary Figure 10. Distribution of GFAP^+^ and Iba1^+^ cells in human GBM tissue.**

a) IBA1^+^ cells were found both inside and outside GBM tumor, while GFAP^+^ cells mainly accumulated at the interface of GBM. b) Immunostaining images showing GFAP^+^ expression at the intratumoral and extratumoral regions. Scale bars represent 200 µm (a) and 100 µm (b).

**Supplementary Table 1: Summary of statistical analyses**

| **Fig.** | **Comparison** | **Sample number**  **^(1)^** | **Method**  **^(2)^** | ***P* value** | **95% CI** | **Significance ^(3)^** |
| --- | --- | --- | --- | --- | --- | --- |
| 1e | PT vs AT | 10 vs 10 | t-test | <0.0001 | -6.972 to -3.961 | Y |
| 1h | IT vs. PT | 9 vs 9 | One-way ANOVA | <0.0001 | -31.43 to -12.13 | Y |
|  | IT vs. AT | 9 vs 9 |  | 0.0205 | -20.87 to -1.575 | Y |
|  | PT vs. AT | 9 vs 9 |  | 0.0301 | 0.9082 to 20.20 | Y |
| 1i | IT vs. AT | 9 vs 9 | One-way ANOVA | 0.0014 | 7.643 to 32.80 | Y |
|  | PT vs. AT | 9 vs 9 |  | 0.0011 | 8.088 to 33.25 | Y |
| 1j | +0-100 vs +100-200 | 10 vs 10 | One-way ANOVA | 0.0002 | 2.943 to 9.443 | Y |
|  | +0-100 vs +300-400 | 10 vs 10 |  | <0.0001 | 4.246 to 10.75 | Y |
| 2c | -GBM vs +GBM (for CD86) | 3 vs 3 | t-test | 0.0006 | 1.571 to 2.789 | Y |
|  | -GBM vs +GBM (for CD206) | 3 vs 3 | t-test | <0.0001 | 4.670 to 5.390 | Y |
| 2d | For GM-CSF: |  | One-way ANOVA |  |  |  |
|  | MG vs GBM | 2 vs 2 |  | 0.0011 | -13785 to -8076 | Y |
|  | GBM vs GBM MG | 2 vs 2 |  | 0.0063 | 3172 to 8882 | Y |
|  | For IL-6: |  | One-way ANOVA |  |  |  |
|  | MG vs GBM | 2 vs 2 |  | <0.0001 | -23285 to -19231 | Y |
|  | GBM vs GBM MG | 2 vs 2 |  | 0.0956 | -465.1 to 3589 |  |
|  | For G-CSF: |  | One-way ANOVA |  |  |  |
|  | MG vs GBM | 2 vs 2 |  | 0.0002 | -26945 to -19500 | Y |
|  | GBM vs GBM MG | 2 vs 2 |  | 0.0053 | 4638 to 12083 | Y |
| 2e | GBM vs GBM MG | 3 vs 3 | One-way ANOVA | <0.0001 | -188.9 to -113.2 | Y |
|  | MG vs GBM MG | 3 vs 3 |  | <0.0001 | -189.8 to -114.1 | Y |
|  | (GBM CM + MG) vs (GBM CM + MG + aG-CSF) | 4 vs 4 |  | 0.0262 | 2.543 to 68.13 | Y |
|  | (GBM CM + MG) vs (GBM CM + MG + aG-CSF + aIL-6 +aGM-CSF) | 4 vs 4 |  | 0.0425 | 0.6504 to 66.24 | Y |
| 3c | -Glutamate vs +Glutamate | 4 vs 4 | One-way ANOVA | 0.0014 | -2.566 to -0.5960 | Y |
|  | +Glutamate vs +Glutamate+KDS2010 | 4 vs 4 |  | 0.0104 | 0.2624 to 2.233 | Y |
|  | +Glutamate vs +Glutamate+TBOA+KDS2010 | 4 vs 4 |  | 0.0006 | 0.7448 to 2.715 | Y |
| 3d | -Glutamate vs +Glutamate | 6 vs 5 | One-way ANOVA | 0.0424 | -1.974 to -0.02597 | Y |
|  | +Glutamate vs +Glutamate+TBOA | 5 vs 5 |  | 0.049 | 0.003393 to 2.038 | Y |
|  | +Glutamate vs +Glutamate+TBOA+KDS2010 | 5 vs 5 |  | 0.0168 | 0.1731 to 2.208 | Y |
| 3f | -Glutamate vs +Glutamate | 10 vs 10 | One-way ANOVA | <0.0001 | -3.537 to -1.803 | Y |
|  | +Glutamate vs +Glutamate+TBOA | 10 vs 10 |  | <0.0001 | 1.233 to 2.967 | Y |
|  | +Glutamate vs +Glutamate+KDS2010 | 10 vs 10 |  | <0.0001 | 1.231 to 2.966 | Y |
|  | +Glutamate vs +Glutamate+TBOA+KDS2010 | 10 vs 10 |  | <0.0001 | 1.328 to 3.062 | Y |
| 3g | -Glutamate vs +Glutamate | 3 vs 3 | One-way ANOVA | 0.0052 | -1.477 to -0.2729 | Y |
|  | +Glutamate vs +Glutamate+TBOA+KDS2010 | 3 vs 3 |  | 0.0434 | 0.01671 to 1.220 | Y |
| 3i | -KDS2010 vs +KDS2010 | 6 vs 6 | t-test | <0.0001 | -0.5916 to -0.4831 | Y |
| 3k | -KDS2010 vs +KDS2010 | 6 vs 6 | t-test | 0.0016 | -0.6052 to -0.1907 | Y |
| 4c | For +GBM: |  | One-way ANOVA |  |  |  |
|  | D2 vs D4 | 3 vs 3 |  | 0.0051 | -6.634 to -1.682 | Y |
|  | D0 vs D4 | 3 vs 3 |  | 0.0015 | -7.756 to -2.805 | Y |
|  | For +GBM+MG: |  | One-way ANOVA |  |  |  |
|  | D2 vs D4 | 3 vs 3 |  | 0.0107 | -2.620 to -0.4700 | Y |
|  | D0 vs D4 | 3 vs 3 |  | 0.0021 | -3.220 to -1.070 | Y |
| 4e | Control vs +TBOA | 3 vs 3 | One-way ANOVA | 0.0008 | 0.1882 to 0.5499 | Y |
|  | Control vs +KDS2010 | 3 vs 3 |  | <0.0001 | 0.4025 to 0.7642 | Y |
|  | Control vs +TBOA+KDS2010 | 3 vs 3 |  | <0.0001 | 0.5275 to 0.8892 | Y |
|  | +TBOA vs +KDS2010 | 3 vs 3 |  | 0.022 | 0.03345 to 0.3951 | Y |
|  | +TBOA vs +TBOA+KDS2010 | 3 vs 3 |  | 0.0014 | 0.1585 to 0.5201 | Y |
| 4f | Control vs +KDS2010 | 3 vs 3 | One-way ANOVA | 0.0194 | -0.9749 to -0.09420 | Y |
|  | Control vs +TBOA+KDS2010 | 3 vs 3 |  | 0.0016 | -1.250 to -0.3691 | Y |
| 4i | -KDS2010 vs +KDS2010 (D14) | 5 vs 5 | Two-way ANOVA | >0.9999 | -14.27 to 12.99 | N |
|  | -KDS2010 vs +KDS2010 (D28) | 5 vs 5 |  | 0.0012 | -37.02 to -9.760 | Y |
| 4j | -KDS2010 vs +KDS2010 | 5 vs 5 | Log-rank (Mantel-Cox) test | 0.0174 | - | Y |
| 5d | Control vs +TBOA | 3 vs 3 | One-way ANOVA | 0.0282 | -5.572 to -0.3363 | Y |
|  | Control vs +KDS2010 | 3 vs 3 |  | 0.0129 | -6.043 to -0.8076 | Y |
|  | Control vs +TBOA+KDS2010 | 3 vs 3 |  | 0.01 | -6.204 to -0.9685 | Y |
| 5e | D4 vs D10 (for +TMZ) | 3 vs 3 | t-test | 0.0051 | -0.7386 to -0.2467 | Y |
| 5f | +TMZ vs +TMZ+KDS2010 | 3 vs 3 | One-way ANOVA | 0.0387 | 0.01027 to 0.3697 | Y |
|  | +TMZ+TBOA vs +TMZ+TBOA+KDS2010 | 3 vs 3 |  | 0.0387 | 0.01027 to 0.3697 | Y |
|  | +TMZ vs +TMZ+TBOA+KDS2010 | 3 vs 3 |  | 0.0017 | 0.1503 to 0.5097 | Y |
| 5j | Vehicle vs +TMZ | 5 vs 5 | One-way ANOVA | 0.0097 | 485795230 to 3852644770 | Y |
|  | Vehicle vs +TMZ+TBOA | 5 vs 5 |  | 0.007 | 580203230 to 3947052770 | Y |
|  | Vehicle vs +TMZ+KDS2010 | 5 vs 5 |  | 0.0075 | 561415230 to 3928264770 | Y |
|  | +TMZ vs +TMZ+TBOA | 5 vs 5 |  | 0.9985 | -1589016770 to 1777832770 | N |
|  | +TMZ vs +TMZ+KDS2010 | 5 vs 5 |  | 0.9992 | -1607804770 to 1759044770 | N |
|  | +TMZ+TBOA vs +TMZ+KDS2010 | 5 vs 5 |  | >0.9999 | -1702212770 to 1664636770 | N |
| 5k | Vehicle vs +TMZ | 5 vs 5 | Log-rank (Mantel-Cox) test | 0.00189 | - | Y |
|  | Vehicle vs +TMZ+TBOA | 5 vs 5 |  | 0.00189 |  | Y |
|  | Vehicle vs +TMZ+KDS2010 | 5 vs 5 |  | 0.00189 |  | Y |
|  | +TMZ vs +TMZ+KDS2010 | 5 vs 5 |  | 0.13429 |  | N |
|  | +TMZ+TBOA vs +TMZ+KDS2010 | 5 vs 5 |  | 0.31731 |  | N |
| ^(1)^ The number of mice used for in vivo experiments were presented as Figure (# of mice) as followed: Figure 1f-j (6), Figure 3h-i (6), Figure 3j-k (6), Figure 4h-j (10), Figure 5g (4), Figure 5h-k (20). Total number of animals used for all in vivo experiments in this study were 52. The sample number for in vitro experiments were presented as biological replication. ^(2)^ Tukey post-hoc one-way ANOVA was performed for comparison between multiple groups. Two-way ANOVA with Bonferroni post-hoc test was used to compare between groups with two independent variables. Two-tailed unpaired Student's *t*-test was performed for two comparisons. Log-rank (Mantel-Cox) test was used to compared difference in survival rate.  ^(3)^ *p* < 0.05 represented significance. | | | | | | |

**Supplementary Table 2: Summary of outcome measures**

| **Figure** | **Method** | **Outcome measure** |
| --- | --- | --- |
| 1b | Imaging | Cell migration |
| 1c | Confocal imaging | Cell distribution and density |
| 1d-e | Imaging | Cell morphology |
| 1f,h,i | Imaging | Cell distribution and density |
| 1g,j | Imaging | Cell morphology |
| 2b,c | Immunostaining | Molecular marker |
| 2d | Cytokine assay | Cytokine expression |
| 2e | Glutamate determination | Glutamate concentration |
| 3b-d | Western blot | Molecular marker |
| 3e-f | Immunostaining | Molecular marker |
| 3g | ELISA | Molecular marker |
| 3h-i | Immunostaining | Molecular marker |
| 3j-k | Immunostaining | Molecular marker |
| 4b-c | Imaging | Tumor size |
| 4d-i | Imaging | Cell distribution and density |
| 4e | Confocal imaging | Cell distribution and density |
| 4d-ii and 4f | Imaging | Tumor size |
| 4h-i | MRI | Tumor size |
| 4j | Viability | Mice survival |
| 5b,d | Imaging | Drug infiltration |
| 5c,e | Imaging | Tumor size |
| 5f | Imaging | Tumor size |
| 5g | IVIS | Drug infiltration |
| 5i-j | IVIS | Tumor size |
| 5k | Viability | Mice survival |
